# Long-term impact of a ten-year intervention program on human and canine *Trypanosoma cruzi* infection in the Argentine Chaco

**DOI:** 10.1371/journal.pntd.0009389

**Published:** 2021-05-12

**Authors:** Marta Victoria Cardinal, Gustavo Fabián Enriquez, Natalia Paula Macchiaverna, Hernán Darío Argibay, María del Pilar Fernández, Alejandra Alvedro, María Sol Gaspe, Ricardo Esteban Gürtler

**Affiliations:** 1 Laboratorio de Eco-Epidemiología, Facultad de Ciencias Exactas y Naturales, Universidad de Buenos Aires, Buenos Aires, Argentina; 2 CONICET-Universidad de Buenos Aires, Instituto de Ecología, Genética y Evolución de Buenos Aires (IEGEBA), Buenos Aires, Argentina; 3 Paul G. Allen School for Global Health, Washington State University, Pullman, Washington, United States for America; Federal University of Ceará, Fortaleza, Brazil, BRAZIL

## Abstract

**Background:**

Interruption of domestic vector-borne transmission of *Trypanosoma cruzi* is still an unmet goal in several American countries. In 2007 we launched a long-term intervention program aimed to suppress house infestation with the main domestic vector in southern South America (*Triatoma infestans)* and domestic transmission in Pampa del Indio, a resource-constrained, hyperendemic municipality with 1446 rural houses inhabited by Creole and indigenous people, in the Argentine Chaco ecoregion. Here, we assessed whether the 10-year insecticide-based program combined with community mobilization blocked vector-borne domestic transmission of *T*. *cruzi* to humans and dogs.

**Methods:**

We carried out two municipality-wide, cross-sectional serosurveys of humans and dogs (considered sentinel animals) during 2016–2017 to compare with baseline data. We used a risk-stratified random sampling design to select 273 study houses; 410 people from 180 households and 492 dogs from 151 houses were examined for antibodies to *T*. *cruzi* using at least two serological methods.

**Results:**

The seroprevalence of *T*. *cruzi* in children aged <16 years was 2.5% in 2017 (i.e., 4- to 11-fold lower than before interventions). The mean annual force of child infection (λ) sharply decreased from 2.18 to 0.34 per 100 person-years in 2017. One of 102 children born after interventions was seropositive for *T*. *cruzi*; he had lifetime residence in an apparently uninfested house, no outside travel history, and his mother was *T*. *cruzi*-seropositive. No incident case was detected among 114 seronegative people of all ages re-examined serologically. Dog seroprevalence was 3.05%. Among native dogs, λ in 2016 (1.21 per 100 dog-years) was 5 times lower than at program onset. Six native adult dogs born after interventions and with stable lifetime residence were *T*. *cruzi*-seropositive: three had exposure to *T*. *infestans* at their houses and one was an incident case.

**Conclusions:**

These results support the interruption of vector-borne transmission of *T*. *cruzi* to humans in rural Pampa del Indio. Congenital transmission was the most likely source of the only seropositive child born after interventions. Residual transmission to dogs was likely related to transient infestations and other transmission routes. Sustained vector control supplemented with human chemotherapy can lead to a substantial reduction of Chagas disease transmission in the Argentine Chaco.

## Author summary

Interruption of vector-borne *T*. *cruzi* transmission is still an unmet goal through the Americas. We launched a long-term intervention program with this goal in rural Pampa del Indio, a resource-limited hyperendemic municipality inhabited by Creole and indigenous (Qom) families in the Argentine Chaco region. The program included blanket insecticide spraying at the outset, sustained vector surveillance including focal insecticide spraying, and community mobilization. Ten years after program onset, we assessed the impacts of interventions on domestic transmission to humans and dogs, taken as sentinel animals. We found that the frequency of human or canine cases decreased dramatically relative to baseline values. No human incident case was recorded. The only infected child born after program onset was attributed to congenital (i.e., vertical) transmission. The few canine cases detected were most likely related to transient domestic infestations and other transmission routes. These results, combined with the quasi-elimination of *T*. *infestans*, support that vector-borne transmission of *T*. *cruzi* to humans had been stopped and this status sustained after interventions.

## Introduction

Chagas disease, a lifelong parasitic infection caused by the protozoan *Trypanosoma cruzi*, is still a major leading cause of disability and premature death in the Americas [1]. Human *T*. *cruzi* infection is mainly transmitted by contamination with feces of triatomine bugs, though congenital (vertical) and oral transmission have gained increasing importance [2]. Originally endemic to the Americas, Chagas disease is currently distributed worldwide hand in hand with the intensified global migration of *T*. *cruzi*-infected people [3]. The Gran Chaco region, encompassing sections of Bolivia, Paraguay and Argentina, is the core area of the main domestic vector in the Southern Cone countries, *Triatoma infestans*. Argentina led the ranking of infected people in the Americas as of 2010 [4].

In the absence of effective vaccines, the main strategy to control vector-borne transmission of *T*. *cruzi* has relied on suppressing domestic triatomines by means of residual insecticide spraying [5,6]. The Southern Cone Initiative was launched in the 1990s to interrupt vector-borne transmission through house spraying with pyrethroid insecticides in endemic areas of Argentina, Chile, Uruguay, Bolivia and Paraguay [7,8]. Measures to block transfusional transmission and via organ transplants were prompted. Over the next 30 years, Chile, Uruguay, Brazil and Paraguay certified the interruption of domestic *T*. *cruzi* transmission mediated by *T*. *infestans* [9]. Control of blood and organ banks has been efficiently established. However, large sections of the Gran Chaco region still face vector-borne transmission; progress was made at smaller geographical scales, such as departments or provinces. In Argentina, 10 of the 19 endemic provinces (i.e., nearly half of the territory is considered endemic) have certified the interruption of domestic transmission mediated by *T*. *infestans* [10].

The domestic transmission cycle of *T*. *cruzi* mainly includes humans, dogs, cats and rodents. Dogs play a crucial role as domestic reservoir hosts of *T*. *cruzi* in many regions through the Americas [11]. Humans cohabiting with infected dogs had a greater risk of *T*. *cruzi* infection than those who did not, and thus infected dogs were an important risk factor for human infection [12,13]. Canine cases preceded human infections with *T*. *cruzi* in northwest Argentina in the context of house reinfestation and transmission resurgence [14]. Dogs have increasingly been used as sentinels of (peri)domestic transmission of *T*. *cruzi* across the Americas [11,15–23]. In rural areas, domestic dogs frequently roam into sylvatic areas and may become exposed to other sources of infection than humans. Regardless of the origin of the infection, the occurrence of *T*. *cruzi*-infected dogs poses a risk to humans cohabiting with them in case house infestation occurs. Therefore, the occurrence of dog infection can be used to stratify domestic transmission risk and to identify high-priority areas for intervention [24], and may provide a bridge between sylvatic and domestic transmission cycles [25].

As part of a long-term program on the eco-epidemiology and control of Chagas disease in the Argentine Chaco region, in 2007 we launched a long-term intervention program based on sustained, supervised vector control supplemented with diagnosis and etiological treatment of children in Pampa del Indio municipality. Program goals were eliminating *T*. *infestans* from rural houses, interrupting domestic *T*. *cruzi* transmission and decreasing disease burden. House infestation with *T*. *infestans* decreased rapidly from 18.0–45.9% to 0.7–12.3% after community-wide spraying with pyrethroid insecticide, though the pace strongly differed among rural sections the municipality (i.e., Areas I-IV) [26–30]. Human seroprevalence with *T*. *cruzi* ranged from 29.0% in Area III (mainly inhabited by an indigenous people, the Qom) [31] to 39.8% in Area I, with a Creole majority [13]. The domestic abundance of *T*. *cruzi*-infected *T*. *infestans* at baseline and cohabiting with ≥3 seropositive people were significant risk factors for human infection [13,31]. The prevalence of *T*. *cruzi* infection in Area I dogs and cats was 26.0–28.7% at program onset [24]. Periodic monitoring of house infestation combined with focal insecticide spraying of reinfested houses over the surveillance phase led to the quasi-elimination of *T*. *infestans* in rural dwellings over 2013–2016 [27,32].

Here, we assessed the occurrence of domestic transmission of *T*. *cruzi* to humans and dogs after a decade of program onset. The key question is whether domestic vector-borne transmission was interrupted throughout the municipality despite of inadequate housing favoring triatomine infestation and chronic resource deprivation. Based on previous findings on the impact of sustained vector control actions on domestic *T*. *cruzi* transmission in the Argentine Chaco [14,16–17] and observed house infestation levels over time, we hypothesized that: i) no human incident case would occur among *T*. *cruzi*-seronegative local dwellers with stable residence; ii) seroprevalence would strongly drop in children under 16 years of age compared with pre-intervention data; iii) *T*. *cruzi*-infected children born after program onset would be more compatible with congenital rather than vector-borne transmission; and iv) native dogs born after interventions and having permanent local residence would rarely be infected with *T*. *cruzi* compared with dogs born before interventions [24]. The results of this study provide relevant information to the multi-country initiatives targeting the interruption of transmission of human *T*. *cruzi* infection of the actual chances of stopping transmission in a hyperendemic scenario.

## Materials and methods

### Ethics statement

This study complied with guidelines on research and biological testing activities involving live vertebrate animals issued by the Institutional Animal Care and Use Committee at the Faculty of Exact and Natural Sciences of the University of Buenos Aires, which is based on the International Guiding Principles for Biomedical Research Involving Animals developed by the Council for International Organizations of Medical Sciences. The research program involving humans and animals was supervised and approved by Comité de Etica en Investigación Clínica “CEIC” from Buenos Aires, Argentina (Protocol No. TW-01-004). All individuals participating in serosurveys or their parents or guardians provided written informed consent. As part of the ongoing cooperation with the local hospital at Pampa del Indio and following national guidelines, people ≤ 18 years of age seropositive for *T*. *cruzi* were referred for etiological treatment.

### Study area

**Fig 1:**
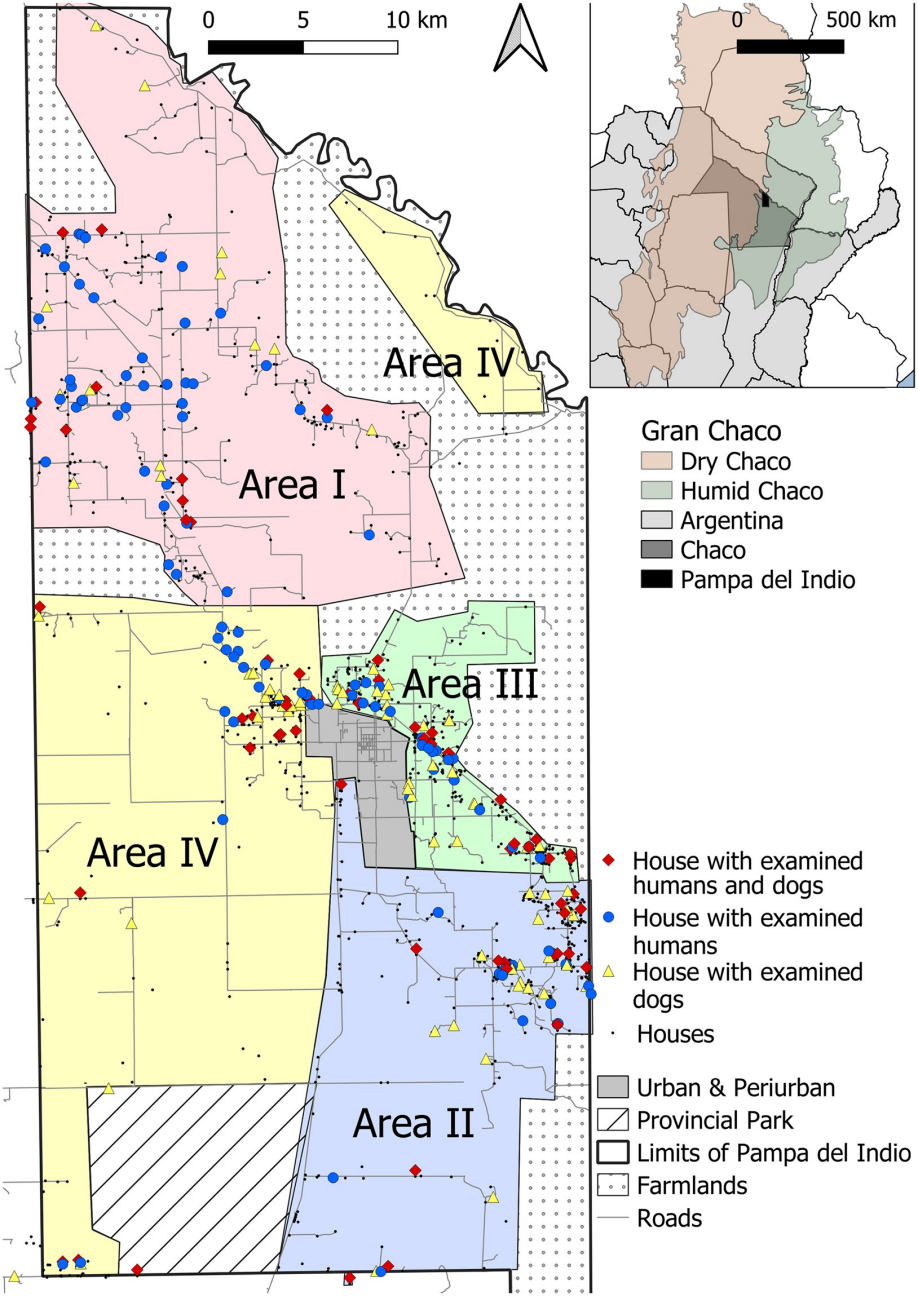
Location of study households included in the human and dog serosurveys. Operational areas are shown as colored-shaded polygons, 2016–2017. Inset shows the location of Pampa del Indio Municipality within the Chaco Province and the Gran Chaco ecoregion. Maps were based on the data collected within the scope of this study and using base layers from Instituto Geográfico Nacional (Argentina). Available online: https://www.ign.gob.ar/NuestrasActividades/InformacionGeoespacial/CapasSIG.

Fieldwork was conducted in rural areas of the municipality of Pampa del Indio (30 by 60 km), in Chaco Province, Argentina. This municipality is located in the transition zone between the dry and humid Chaco (Fig 1). It is subjected to wide fluctuations in annual rainfall, periodic extraordinary flooding and droughts, and is a mosaic of humid and dry Chaco forests [33]. The rural section comprised 1446 inhabited households as of 2016; for operational purposes they were divided in four areas (Areas I-IV) having 300–400 houses each (Fig 1). These areas comprised Qom and Creole descendants almost equitably, though their relative frequency differed between areas. The municipality ranks among the top levels of unmet basic needs in Argentina; structural poverty is chronic. Detailed descriptions of each area can be found elsewhere [26,28,30]. The study area is described in S1 Text.

### Intervention program

House-to-house surveys were performed to georeference and identify all rural houses with a numbered plaque, and to assess baseline house infestation status and household environmental and sociodemographic features (including number of inhabitants, domestic animals and peridomestic sites). The baseline vector surveys were immediately followed by a community-wide insecticide spraying of all premises conducted by government-sponsored vector control teams (S1 Fig). Field operations began in Area I in late 2007 and were gradually scaled up to the rest of the municipality until achieving full coverage of all rural sections in 2010. Insecticide spraying was followed by community-based vector surveillance and periodic vector surveys performed in cooperation between the research team and vector control personnel [26–30,34]. We implemented community meetings and workshops at local primary healthcare posts, churches and schools to mobilize householders and increase awareness on their crucial role in vector surveillance and disease prevention. Additional workshops were organized before each human serosurvey and etiological treatment round as described [35]. Etiological treatment was initially offered to *T*. *cruzi*-seropositive children ≤18 years of age, and was subsequently scaled up to those aged ≤21 years and women in child-bearing age. We did house-to-house visits in search of eligible patients and to enhance adherence to serodiagnosis and etiological treatment; organized community workshops to explain the pros and cons of chemotherapy, and offered decentralized serodiagnosis and treatment as described [35].

### Study design

Cross-sectional serosurveys were conducted house-to-house in June-July 2016 (dogs) and June, August, September, December 2017 and April 2018 (humans). For both human and dog serosurveys we used a stratified sampling design based on the house-level occurrence of *T*. *infestans* during the vector surveillance phase (2008–2016). Venipuncture was performed by trained veterinarians (dogs) or healthcare personnel from the local hospital (humans).

#### Humans

We used the information collected during previous human serosurveys conducted over 2011–2016 (achieving almost 50% coverage of nearly 9000 rural inhabitants) for risk-stratum definition [13,36,37]. In order to maximize the chance of finding human incident cases, we included all medium- and high-risk houses for domestic *T*. *cruzi* transmission defined as follows. High-risk houses were all those ever found infested with *T*. *infestans* (i.e., at least once) by timed-manual searches during the surveillance phase. Medium-risk houses were those with high domestic abundance of *T*. *cruzi*-infected *T*. *infestans* at baseline (i.e., ≥9 infected bugs by timed-manual searches) or households comprising ≥3 seropositive people but with no *T*. *infestans* collected during the surveillance phase. Low-risk houses were those that did not fulfill the above-mentioned criteria. We randomly selected one low-risk house every two high- or medium-risk houses. In total, we listed and visited 129 inhabited high-risk, 83 medium-risk and 96 low-risk houses, of which 59 (46%), 35 (42%) and 42 (44%) were effectively surveyed (S2 Fig). The low-risk sample also included 44 additional households where people lacked a Chagas serodiagnosis or who required new testing to confirm individual serostatus. The selected households that did not participate in the human serosurvey harbored residents who refused a repeat venipuncture (n = 97), had moved away (n = 20), were busy at that time (n = 5), absent (n = 32) or had no eligible resident (n = 18) (see below).

Children born after the initial insecticide spraying campaign and *T*. *cruzi*-seronegative people were eligible for venipuncture at each selected house. Eligible persons or their parents or guardians (for children) signed a written consent. People seropositive for *T*. *cruzi* were not eligible to avoid repeated venipuncture given that spontaneous seroreversion of *T*. *cruzi* infection rarely occurs. Seropositive individuals who had been treated with benznidazole or nifurtimox were not eligible for present purposes; their serostatus was monitored as part of an ongoing research effort and the outcome used for seroprevalence estimation. Eighteen selected households contained no eligible people (i.e., minors without guardians, diseased people or only seropositive people). A short questionnaire was administered to each person at the time of venipuncture to register their complete names, identification document number, date of birth, gender, parents’ names, parents’ prior serodiagnostic results for Chagas disease (if known), current residence (i.e., house identification number and name of the head of household), residence history (emphasizing on local stable residence and occasional travel outside), previous Chagas serodiagnostic results (if any, i.e., self-reported serostatus), and blood transfusion history.

#### Dogs

Rural dog populations in the Argentine Chaco have a short mean life expectancy of approximately 3 years [11]. Therefore, we narrowed the time frame for household risk stratification to the previous five years (i.e., 2011–2016) to maximize the chance of finding dogs that might have been exposed to *T*. *infestans*. Houses infested with *T*. *infestans* at any time over this period were considered to be of high-risk of vector-borne transmission. Therefore, all 77 houses (representing 5.3% of all inhabited houses) where *T*. *infestans* was detected over 2011–2016 were eligible for blood testing. An equal number of inhabited houses negative for *T*. *infestans* over 2011–2016 (i.e., low-risk houses) were randomly selected. When the selected low-risk house was closed at the time of the survey, the nearest inhabited low-risk house was surveyed. Nearly a third (29.9%) of high-risk houses eligible for the dog serosurvey no longer existed, were vacant or closed at the time of the serosurvey, whereas 10.8% of low-risk houses were in such status. One high-risk house only harbored a puppy and therefore was not eligible for examination. Thus, the participating dogs belonged to 53 high-risk and 98 low-risk houses, respectively (additional low-risk houses were added on a self-demand basis) (S2 Fig). No owner refused to have their dogs tested. All dogs >3 months of age were eligible for venipuncture. In total, 73 eligible dogs were absent or too aggressive to be examined. We took a picture of each dog for future identification, and completed a questionnaire addressing each animal’s name, owner, village and house code, particular features, food habits, function, origin, stable local residence, and female fecundity history. This thorough interview to dog owners provides key information to eventually distinguish among local domestic transmission, local sylvatic transmission and imported cases.

#### Serology

Each individual was tested by at least two serological methods. Human anti-*T*. *cruzi* antibodies were detected using two ELISAs that either included *T*. *cruzi* epimastigote lysate or recombinant antigens (Chagatest, Wiener, and ELISA Rec V3.0, Wiener, respectively) as in our previous human serosurveys [13,35–37]. Their sensitivity and specificity were 98.8%-100% and 98.7%-100%, respectively, according to the manufacturer and an international evaluation [35,38]. Samples with an optical density deviation of 10% above the cut-off were considered positive, whereas those that were 10% below the cut-off were considered negative. Dog sera were tested with an in-house enzyme-linked immunosorbent assay (ELISA) and a commercial IHA test (Wiener Laboratories S.A.I.C., Buenos Aires, Argentina) as described elsewhere [39]. Each serum was tested in duplicate in ELISA assays. A human or dog serum sample was considered seropositive for *T*. *cruzi* when it was reactive for two tests. Eleven serologically discordant human samples were sent to the National Institute of Parasitology “Dr. Mario Fatala Chabén” (ANLIS-Malbrán, Buenos Aires, Argentina) for final serodiagnosis where they were tested by IHAT, ELISA and IFAT. The only serologically discordant dog sample was also tested by indirect immunofluorescence (IFAT) at Laboratorio DIAP (Argentina).

#### Xenodiagnosis

Seropositive dogs were examined by xenodiagnosis in March 2017 to confirm *T*. *cruzi* infection and isolate parasites. A total of 20 uninfected, laboratory-reared third- or fourth- instar nymphs of *T*. *infestans* were applied directly to the belly of each dog for 20 minutes. Xenodiagnostic bugs were microscopically examined (400×) 30 and 60 days after exposure [39].

### Data analysis

This manuscript complies with the STROBE checklist (S2 Text). Household data on human and dog infection were merged with the triatomine database. Agresti-Coull 95% confidence intervals were calculated for seroprevalence. We used Wilson 95% confidence intervals when the number of examined subjects was ≤ 40 [40].

For humans, each individual’s age at the date of the serosurvey was back-corrected (as explained in [31]) to represent the age each person had at baseline (before interventions). The rationale for doing so was double: to reflect duration of past exposure to vector-borne transmission, and to allow comparison between observed seroprevalence rates and previous data from Areas I-IV (which differed in timing of blanket insecticide spraying). This assumed that exposures occurring after interventions were negligible in comparison with exposures occurring before interventions. Information on age, ethnic background and history of travel outside the study area was missing for three, two, and 21 persons, respectively.

Dogs were classified according to their origin as urban immigrants if they came from Pampa del Indio town or other city; rural immigrants if they came from other rural villages outside of Pampa del Indio municipality or if the dog was a stray; and natives if they were born in any rural village within Pampa del Indio municipality. As our sample was enriched in high-risk houses (which represented a minority in the whole municipality), we adjusted the observed *T*. *cruzi* prevalence in dogs by taking into account the municipality-level fraction of low- and high-risk houses. We compared the date of house infestation occurrence at the dog’s house with each dog’s age to estimate whether each individual had been exposed to triatomines and for how long. One seronegative dog aged one month was mistakenly included in the survey. Information on origin and age was missing for 13 and 16 study dogs, respectively. We examined the house infestation status at the dog’s house of residence and at all inhabited houses within a one km radius (taken as a rough estimate of dog home range [41]) to allow for the fact that most rural dogs were unrestrained and may roam freely. Statistical analyses were run in R (version 3.3.1) [42]. Confidence intervals and univariate odds ratios were estimated using the “binom” [43] and the “lme4” [44] packages, respectively.

We joined the vector and human seropositivity data to evaluate the association between triatomine exposure at the house of residence during the vector surveillance phase and seropositivity for *T*. *cruzi* both in children <16 years of age and in humans who had been examined serologically before. We distinguished between people living in high-risk houses (who were allegedly exposed to triatomines after initial serodiagnosis) from those that were not exposed. Modelling *T*. *cruzi* infection via multiple logistic regressions with random effects was hampered by the finding of only one child case and six infected dogs born after interventions. Thus, a case study analysis was done instead. The instantaneous per capita rate of conversion from negative to positive (mean annual force of infection, λ) in humans and dogs was estimated retrospectively using a catalytic model [13]. λs were estimated using the “minpack.lm” package [45]. To estimate λ before interventions, we included 346 children inhabiting 129 households of Area I [13], 875 children inhabiting 332 households of Area III [31], and 623 children inhabiting 237 households of Area II and IV [37]. Therefore, for pre-intervention calculations, we included 1844 children (with back-corrected age) born before the onset of interventions; in 2017 we included 159 children inhabiting 83 households who were born before or after the onset of interventions. For dogs, we estimated λ retrospectively for 375 animals born before program onset (residing in 176 households of Area I as of 2008 [24]), and for 287 dogs born after program onset as of 2016.

## Results

### Demography

We examined 410 people from 180 households, representing 12% of all rural houses, for *T*. *cruzi* antibodies (Table 1). Their median age was 21 years (range, 0.8–86); 38.8% of them were <16 years of age, and 58.3% were of Qom descent. Nearly half of them inhabited high- (30%) or medium-risk houses (22%) whereas the remainder inhabited low-risk houses (48%).

**Table 1:** Demographic characteristics of human and dog populations, Pampa del Indio, Chaco, 2016–2017.

	Humans		Dogs
Demographic attribute	Examined	Not examined*	Examined
Number (no. of houses)	410 (180)	501 (129)	492 (151)
Median age (range, in years)	21 (0.8–86)	21 (1–87)	2 (0.1–20)
Male-to-female ratio	1.2	1.3	1.5
Born after interventions (%)	25.0	18.8	89.0
Residing in high-risk houses (%)	30.0	24.2	33.9
Qom households (%)	58.6	78.6	65.7
Native resident (%)	NR	NR	66.3
Outside travel history (%)	14.3	NR	0.0

NR: not registered

*Includes reluctant and not eligible individuals

In total, 492 dogs from 151 houses were examined for *T*. *cruzi* (Table 1). The study dogs had a median age of 2 years; most of them (89%) had been born after the onset of interventions. Nearly two-thirds inhabited Qom households and were native to Pampa del Indio rural areas. No dog was reported to have travelled outside of the municipality. Very few households lacked a dog (5.0%), harbored only a pup too young for serodiagnosis (0.5%) or an aggressive dog that could not be examined (1.5%).

### Human seroprevalence

**Fig 2:**
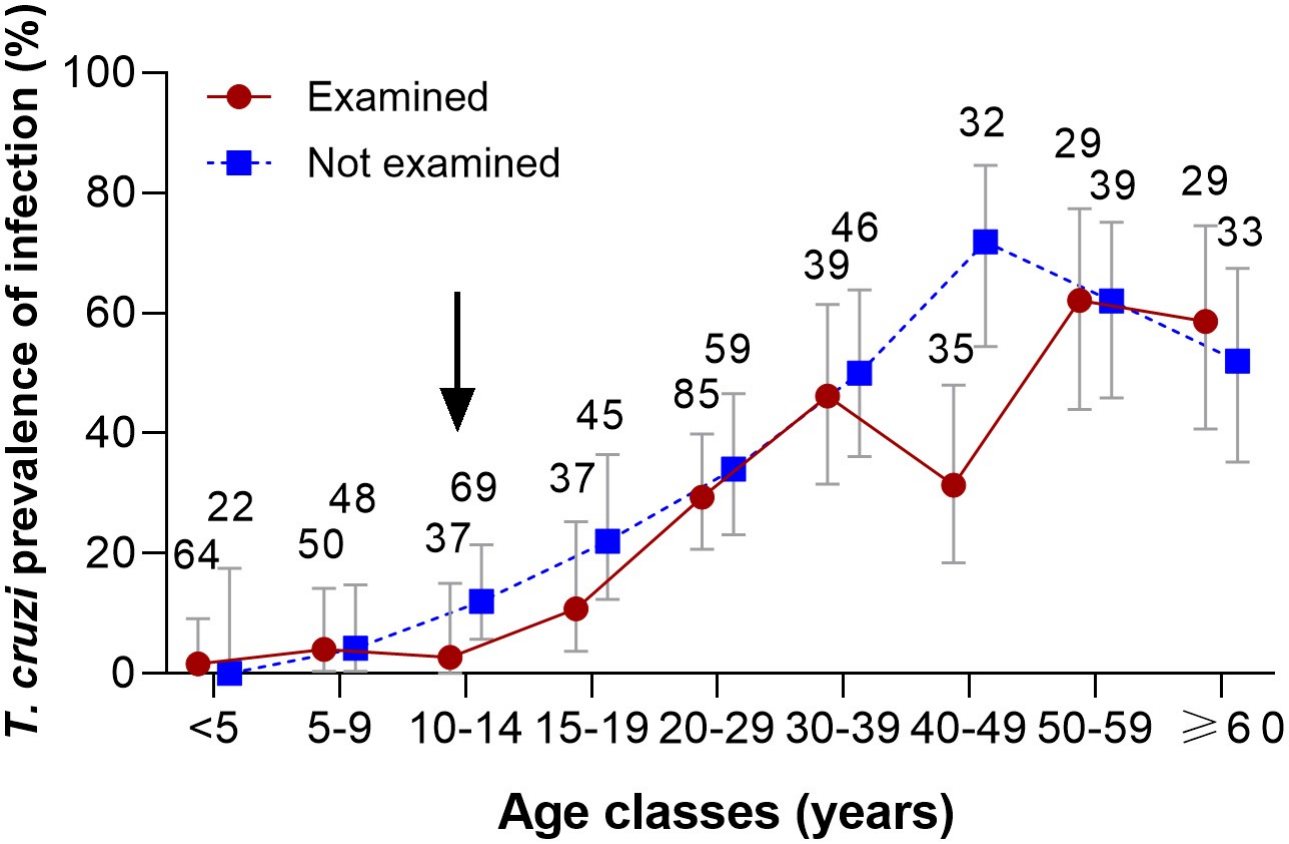
Age-specific seroprevalence of *Trypanosoma cruzi* in humans according to examined and self-reported status, Pampa del Indio, Chaco, 2017. The arrow indicates the occurrence of community-wide insecticide spraying and initiation of vector surveillance. Figures indicate the number of people examined for antibodies or reporting their serostatus for *T*. *cruzi*. Five people with unknown age were excluded.

Nearly a quarter (23.66%; 95% CI = 19.79–28.01%) of 410 tested individuals were seropositive for *T*. *cruzi*. In total, 501 people from the study houses refused to provide a blood sample in 2017, most of which alleged that they had already been tested and their house was free from infestation. Of these, 313 people had previously been tested by us, and other 99 people self-reported their serostatus for *T*. *cruzi*. By combining these data for individuals not examined in 2017, the seroprevalence was 31.07% (95% CI = 26.79–35.70%). The age-seroprevalence curve increased from 1.6% in children aged <5 years to 46.2% in people aged 30–39 years, and ranged between 31.4% and 71.9% in older age groups (Fig 2). Both age-seroprevalence curves were notably similar except in the age group 40–49 years old.

### Child seroprevalence

**Fig 3:**
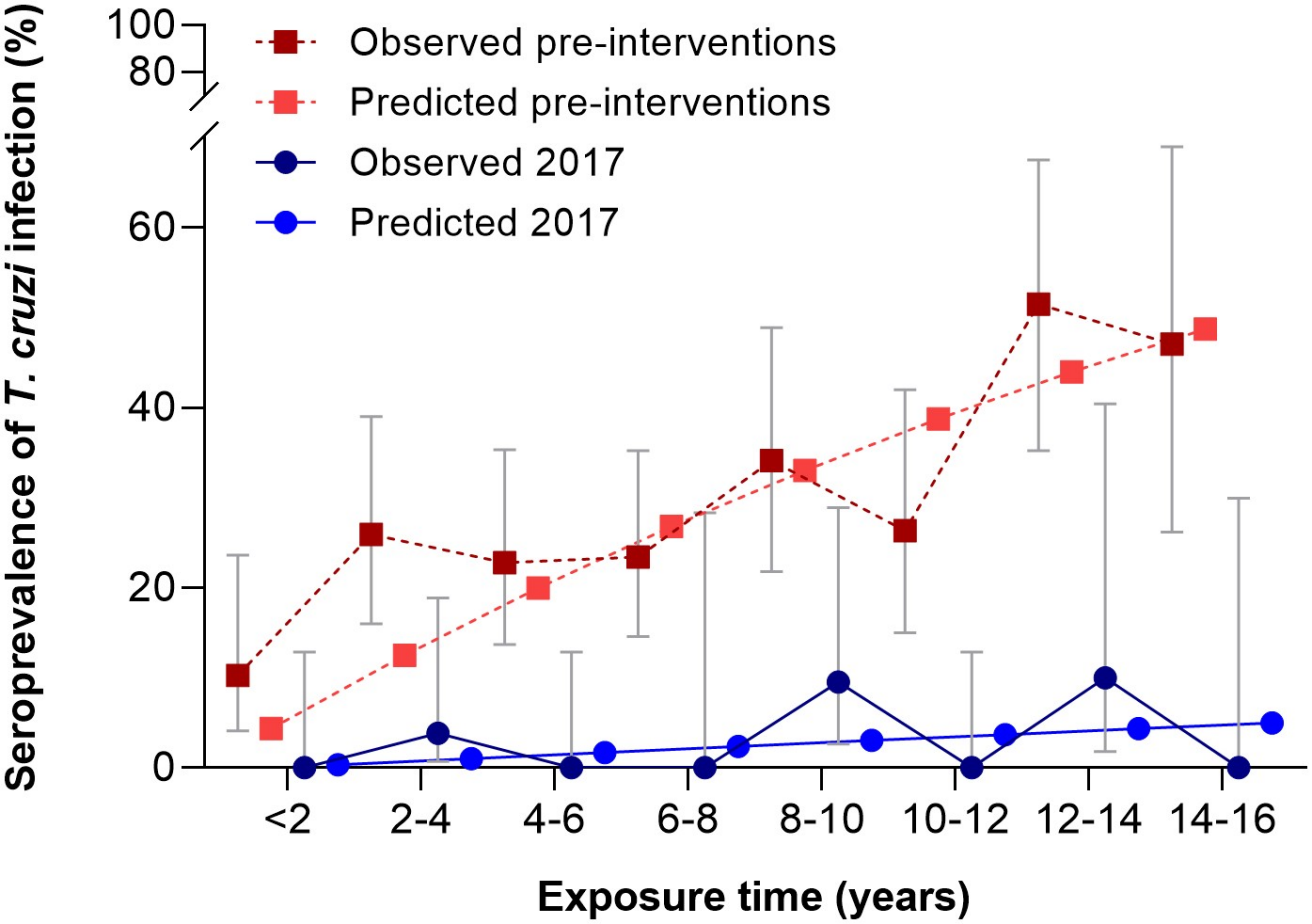
Observed and predicted (irreversible catalytic model) seroprevalence of *Trypanosoma cruzi* in children with less than 16 years of exposure before interventions and in 2017, Pampa del Indio, Chaco.

The seroprevalence of *T*. *cruzi* in children aged <16 years was 2.5% in 2017, i.e., 4- to 11-fold lower than before vector control interventions, when seroprevalence ranged from 10.6% (Area III) to 27.7% (Area I). We estimated the mean annual force of infection (λ) experienced by children aged <16 years before interventions (2007–2008) and in 2017. λ sharply decreased 6 times from 2.18 per 100 person-years (95% CI = 1.89–2.47) before interventions to 0.34 (95% CI = 0.00–0.76) in 2017 (Fig 3). Child seroprevalence in 2017 decreased with increasing house infestation risk (high risk: 1.96%, n = 51; medium risk: 3.71%, n = 54, and low risk: 3.85%, n = 52) but not significantly so (χ^2^ = 0.37, df = 2, *P* = 0.83). This highly unlikely trend may be explained by the very few infested houses recorded during the surveillance phase and the consequently very few people tested in them. There was no association between observed triatomine exposure and *T*. *cruzi* infection in children <16 years of age (Table 2).

**Table 2:** Seroprevalence of *Trypanosoma cruzi* infection in children <16 years of age according to observed exposure to *Triatoma infestans* (unless otherwise noted) during the vector surveillance phase, Pampa del Indio, Chaco, 2008–2016.

	% seropositive (no. examined)		
	Born after (n = 102)	Born before the onset of interventions (n = 57)	
House infestation status	Vector exposure** during surveillance	Vector exposure at baseline	Vector exposure during surveillance
Domestic infestation			
No	1.0 (96)	4.2 (24)	6.8 (44)
Yes	0.0 (6)	0.0 (16)	0.0 (12)
ND/NE*	--	11.8 (17)	0.0 (1)
Peridomestic infestation in nearby habitats^$^			
No	1.0 (98)	2.9 (34)	5.8 (52)
Yes	0.0 (4)	0.0 (6)	0.0 (4)
ND/NE*	--	11.8 (17)	0.0 (1)
Peridomestic infestation in other habitats^&^			
No	1.0 (99)	3.0 (33)	4.4 (45)
Yes	0.0 (3)	0.0 (7)	9.1 (11)
ND/NE*	--	11.8 (17)	0.0 (1)
Presence of *T*. *cruzi*-infected *T*. *infestans*			
No	1.0 (100)	3.1 (32)	5.7 (53)
Yes	0.0 (2)	0.0 (6)	0.0 (3)
ND/NE*	--	10.5 (19)	0.0 (1)
House infestation with *T*. *sordida*			
No	1.1 (89)	2.9 (34)	6.1 (33)
Yes	0.0 (13)	0.0 (6)	4.3 (23)
ND/NE*	--	11.8 (17)	0.0 (1)

** Vector exposure to *T*. *infestans*.

ND/NE* Includes houses not inspected for infestation.

^$^ Nearby peridomestic habitats include kitchens, storerooms and mud ovens.

^&^ Other peridomestic sites mainly include chicken coops and corrals.

### Human incidence of infection

All *T*. *cruzi*-seronegative people examined initially over 2010–2015 and re-examined in 2017 (n = 114) remained seronegative regardless of whether they had any exposure to *T*. *infestans* or not (Table 3).

**Table 3:** *Trypanosoma cruzi* infection and incidence rate among seronegative human residents according to domestic exposure to *T*. *infestans* after initial serodiagnosis, Pampa del Indio, 2017.

House risk level	Exposure to *T*. infestans*	Seroprevalence (no. examined)	Incidence rate per 100 person-years (exposure time)^&^
High	Yes	0 (2)	0 (13.7)
	No	0 (35)	0 (192.3)
Medium	No	0 (30)	0 (147.6)
Low	No	0 (44)	0 (209.8)
Total		0 (114)	0 (563.3)

* After initial serodiagnosis.

^&^ Years lived by the persons in each category, calculated as the sum of time spanned between the initial negative serodiagnosis and re-examination in 2017 for each person.

Only one of 102 children (0.98%, 95% CI = 0.00–5.88%) born after program onset was seropositive to *T*. *cruzi* in 2017. He was born to a *T*. *cruzi*-seropositive mother and lived in an apparently uninfested house both before and after interventions; had no travel record outside of the study area, and did not cohabit with infected dogs. After screening all the human serosurvey data collected for Pampa del Indio municipality over 2010–2017, we found that only 8 (1.6% of 511) children born after program onset were seropositive for *T*. *cruzi*. All of them resided in apparently uninfested houses since birth, and their mothers were *T*. *cruzi*-seropositive (Table 4). Only two completed the 60-day course of etiological treatment despite of repeated offers. The fraction of putative congenital cases born to seropositive mothers was 3.8% (8 of 213) (Table 4). If λ before interventions had remained unchanged at pre-intervention levels, 58 new cases of *T*. *cruzi* infection would have occurred over the average lifetime exposure (5.2 years) of each of the 511 children born after program onset.

**Table 4:** Seropositivity for *Trypanosoma cruzi* infection in children born after the interventions according to maternal serostatus. Pampa del Indio, Chaco.

Child status	Maternal status			
	Seropositive	Seronegative	Unknown	Total
Seropositive	8	0	0	8
Seronegative	205	264	34	503
Total	213	264	34	511

### Dog infection

The overall seroprevalence of *T*. *cruzi* infection in 492 dogs was 3.05% (95% CI = 1.81–5.01%). Nearly a third (33.94%) of all tested dogs resided in high-risk houses (Table 1). The relative odds of dog seropositivity in high-risk houses was 5.66 times higher (6.59%; 95% CI = 3.60–11.63%) than in low-risk houses (1.23%; 95% CI = 0.36–3.24%) (Table 5). Based on the observed seroprevalence of *T*. *cruzi* infection in dogs residing in low- and high- risk houses, we estimated the adjusted prevalence across the municipality (i.e., as if dogs from all inhabited houses had been examined). Low-risk houses comprised the majority (94.7%) of inhabited houses; hence, the adjusted seroprevalence was 1.51% (95% CI = 0.53–3.68%).

**Table 5:** Risk factors for seroreactivity to *Trypanosoma cruzi* infection in dogs, Pampa del Indio, Chaco.

Factor	OR	95% CI	% seropositive (n)
Inhabiting high-risk houses			
No	1	-	1.23 (325)
Yes	5.66	1.77–18.06	6.59 (167)
Born before interventions^&^			
No	1	-	2.28 (438)
Yes	5.94	1.93–18.33	12.20 (41)
Dog origin^&^			
Urban immigrant	1	-	2.88 (104)
Rural immigrant	0.7	0.03–5.64	2.04 (49)
Native	1.18	0.36–5.27	3.37 (326)

^&^ Excludes 13 dogs with no data for this factor.

The odds of seropositivity for *T*. *cruzi* was 5.94 times higher in dogs born before interventions (12.20%, n = 41) than in dogs born after them (2.28%, n = 438) (Table 5). The former accounted for a third of the extant seropositive cases as of 2016. The infected dogs inhabited 13 houses (0.90% of all inhabited houses). Seroprevalence among urban immigrants (2.88%, n = 104), rural immigrants (2.04%, n = 49) and native dogs (3.37%, n = 326) was not significantly different (Pearson χ^2^ test, df = 2, *P* = 0.87). Of the 15 seropositive dogs detected, 8 (53%) survived to March 2017 and were examined by xenodiagnosis; 5 (62.5%) of them were xenodiagnosis-positive.

**Fig 4:**
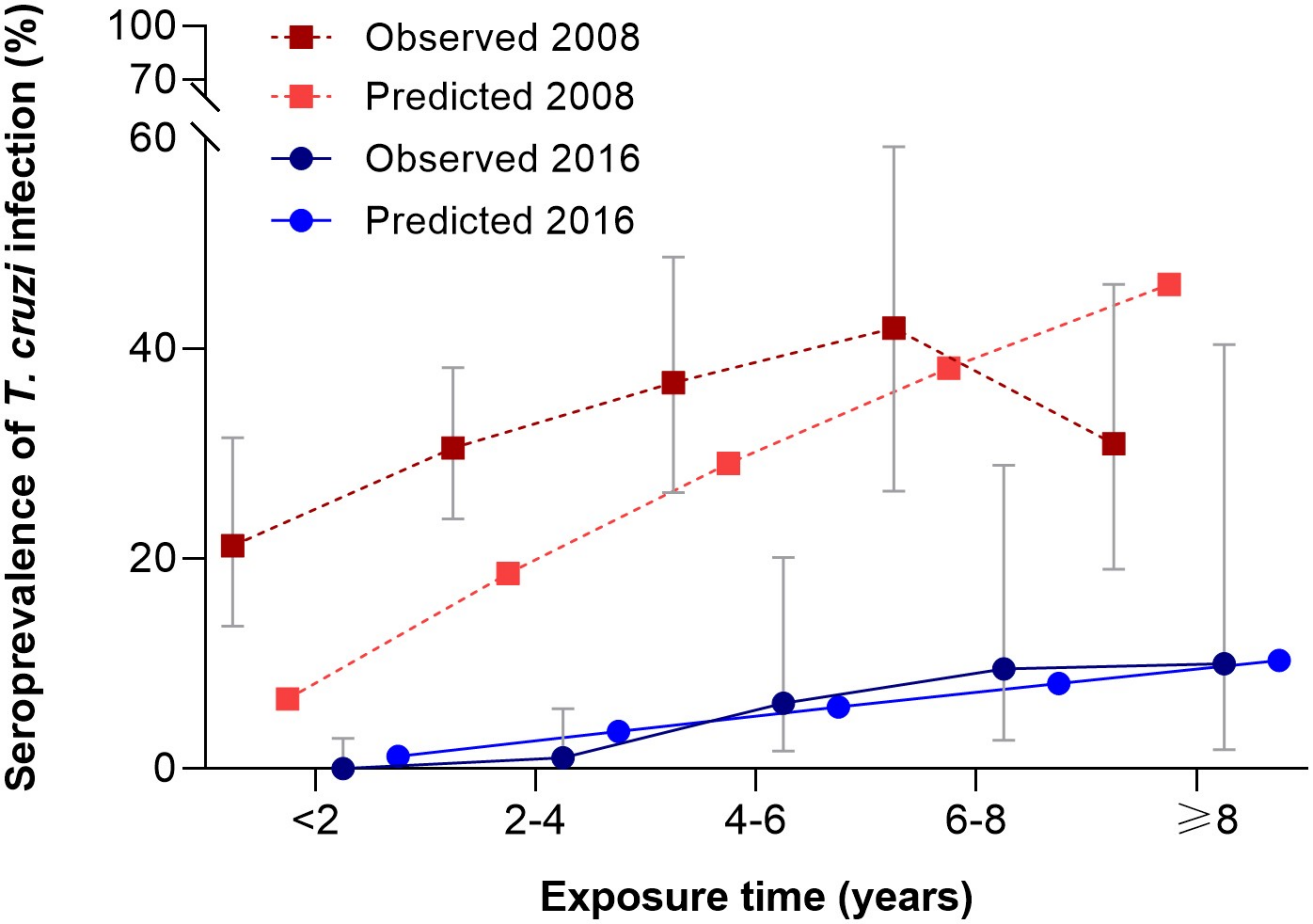
Age-specific seroprevalence of *Trypanosoma cruzi* infection in dogs, Pampa del Indio, Chaco, 2016. Expected seroprevalence derives from the irreversible catalytic model.

Among native dogs, seropositivity for *T*. *cruzi* was nil in those aged <1–2 years, and increased from 1.1% in three-year-old dogs to 9.5–10.0% in those aged 6–7 years or older (Fig 4). Among 287 native dogs born after interventions, the relative odds of seropositivity was nearly 7 times higher in dogs that had been exposed to *T*. *infestans* at their place of residence (7.7%) than in dogs that had not (1.2%) (OR = 6.81, 95% CI: 1.32–35.02).

We estimated the mean annual force of infection (λ) retrospectively for dogs born before program onset, which inhabited 176 households of Area I as of 2008 [24], and for dogs born after program onset as of 2016. In 2008, λ was 6.87 per 100 dog-years (95% CI = 4.7–9.4%) based on five age groups, and seroprevalence was systematically underestimated by the irreversible catalytic model (Fig 4). λ in 2016 was 5 times lower (λ = 1.21 per 100 dog-years, 95% CI = 0.8–1.7%) than in 2008, and the predicted seroprevalence rates fitted closely the observed estimates.

We found 6 *T*. *cruzi*-seropositive, native dogs born after interventions which had stable residence. The serostatus of their mothers was unknown, except for one dog born to a seronegative female. All were hunter dogs and were reported to consume remains of sylvatic mammals, including blood and viscera. Three seropositive dogs (aged 5–7 years old) had lifetime residence in low-risk, apparently uninfested houses (S1 Table). The remaining three seropositive dogs (aged 3, 5 and 8 years old) inhabited high-risk houses where they had been exposed to *T*. *infestans*. One of them was an incident case although no triatomine had been collected at its house or in neighboring ones after testing seronegative in 2013.

## Discussion

Our results support the interruption of vector-borne transmission of *T*. *cruzi* to humans and a sharp drop in the prevalence of infected dogs following community-wide house spraying with insecticide and sustained vector surveillance over a decade in a hyperendemic municipality of the Argentine Chaco. The mean annual forces of infection experienced by both human and dog populations were 5–6 times lower than those that prevailed before interventions. No incident case was detected amongst the seronegative human residents that were re-examined for *T*. *cruzi* antibodies. The only seropositive child born after program onset was most likely attributable to congenital transmission as there was no evidence of exposure to (peri)domestic triatomines. No acute Chagas disease case was detected or reported locally over the follow-up.

The large reduction in domestic transmission in Pampa del Indio is closely and directly related to the documented impacts of insecticide-based control actions on house infestation. The prevalence of domestic infestation with *T*. *infestans* plummeted over the first year after initial community-wide interventions and remained below 5% in Areas I-III over three to seven years after initial interventions, as determined jointly by timed-manual searches with a dislodging aerosol and householders’ bug collections [27,29,30]. An exhaustive inspection of all inhabited rural houses by timed-manual searches in April-May 2016 corroborated that house infestation was less than 1% [45]. The limited sensitivity of timed-manual searches to detect house infestation at low densities [46] was compensated by repeated searches over the extended follow-up and household-based vector surveillance. Had an upsurge of domestic infestation occurred over the surveillance phase in some district section, the chance of failing to reveal it at least once by either bug detection method on repeated search occasions would be marginal.

The seroprevalence of *T*. *cruzi* in children aged ≤5 years has traditionally been used to monitor the impacts of vector control actions on domestic transmission (e.g., [47–49]. In our study, the seroprevalence of *T*. *cruzi* in children aged <5 years was 1.6% (95% CI, 0–9.1%), i.e., three, four and 11 times lower than the respective preintervention seroprevalence in Area III (5.4%), Area II and IV (6.1%), and Area I (17.9%) [13,31,37]. For example, Chile achieved a five-fold decrease in the seroprevalence of *T*. *cruzi* in children aged <5 years to 1.1% after 12 years of nation-wide vector control activities [50]. In Brazil, a nation-wide random survey of children aged ≤5 years yielded a seroprevalence of 0.03% over 2001–2008 after large-scale interventions had been launched in 1975–1980 [48]. Here we considered a broader age group; serological follow-up of seronegative individuals of all ages, and maternal seropositivity status to make inferences on transmission status.

Long-established guidelines for Chagas disease required that vector-borne transmission at a defined administrative scale has been interrupted when seroprevalence in children aged ≤5 years was <2%; no acute cases had been notified to the surveillance system over the previous three years, and domiciliary infestation rates were <1% [47]. Although these criteria may work well with native triatomine species at province- or state-wide scales, there are several caveats to each of the metrics and the defined administrative level to which they apply [51]. Others considered that the most informative child age class to assess transmission interruption remained to be identified [52]. We note that the average duration of exposure of children ≤5 years old in a closed, stable population growing at a fixed rate would be below the mid-point (i.e., 3 yr) of the under-five age class. In practice, infants (<1 yr old) may not be accessible for screening due to lack of parental permission to perform finger prick or venipuncture, or the eventual occurrence of maternally derived antibodies to *T*. *cruzi* may render the outcome uninformative for assessing the occurrence of vector-borne transmission. In strongly seasonal environments, such as those in the Argentine Chaco, transmission seasons span from late winter to early fall when ambient temperatures exceed the triatomines’ activity threshold [53]. Hence, restriction of impact assessments to children aged ≤5 years would at most reflect the short-term effects of control actions on vector-borne transmission over an average of two seasons. Such short-term evaluations can hardly have any predictive power on its own (i.e., when devoid of considerations on the consistency and effectiveness of the vector surveillance system and other local risk factors such as housing conditions, domesticity of the target vector species, and the prevalence of human infection with *T*. *cruzi*).

Interruption of vector-borne transmission is the direct consequence of control actions that suppress the occurrence of the target triatomine species and vector infection in domestic habitats. Host infections with *T*. *cruzi* (human, dog and cat) fuel domestic transmission. Our intervention program, based on sustained, supervised community-based vector surveillance and selective control actions after community-wide spraying with pyrethroid insecticide, was supplemented with diagnosis and etiological treatment of seropositive children under 18 years of age. To achieve these ends, we promoted community empowerment through meetings and workshops, and close collaboration with local public health and school personnel, and other stakeholders. The program did not intend to measure the potential effects of combined serodiagnosis and etiologic treatment on vector-borne transmission. Control actions likely interrupted vector-borne transmission of human infections virtually within the first year of program onset at each operational area, as suggested by residual or nil levels of domestic infestation and bug infection thereafter. This putative time period to transmission interruption is substantially shorter than that predicted by a mathematical model in moderate (5–6 yr) or highly endemic (>10 yr) scenarios [54], which at best only partially match those in the Argentine Chaco where sylvatic triatomine species apparently hardly contribute to domestic transmission (e.g., [55–58]). No sylvatic focus of *T*. *infestans* has been found in the humid Chaco so far, including Pampa del Indio [59], and the occurrence of sylvatic *T*. *infestans* appears to be rare both in disturbed and undisturbed forests of the dry Argentine Chaco [57,60].

Housing improvement or replacement and other parallel socio-demographic processes may facilitate or hinder the interruption of domestic vector-borne transmission. The nomadic roots of Qom households likely diminished human exposure to triatomines in a substantial fraction of the population classified as “movers”, i.e., households that changed the location of their homes within Area III [31]. The exact magnitude of this process varied widely among operational areas. Additionally, a government-based housing program provided brick-and-cement houses less suitable for triatomines. However, this program mainly targeted Qom households in some communities; for example, in Area III, it modestly increased the proportion of houses with brick-and-cement walls from 26% at baseline to 42% at approximately 7 years after interventions [27]. In most cases, households kept both the old mud-and-thatch houses and the new ones.

The role of dogs, cats and children as the main sources of *T*. *cruzi* that drive domestic transmission decreased in parallel to sustained vector control actions through rapid dog and cat population turnover [11] and etiological treatment of seropositive people ≤18 years of age. However, a large number of untreated *T*. *cruzi*-seropositive adults remained as long-term parasite sources, albeit with a lower parasite burden and infectivity to the vector than younger groups [61]. The seroprevalence of human infection with *T*. *cruzi* remained at high levels (23.7–31.1%) in Pampa del Indio after a decade of effective control interventions that disarticulated the strong link between household seroprevalence and current house infestation status (e.g., [62,63]). Similarly, in Arequipa (Peru), human infection with *T*. *cruzi* was associated with past exposure to triatomines and infections originated elsewhere rather than to the local occurrence of *T*. *cruzi*-infected triatomines [64].

Suppression of vector-borne transmission may allow other pathways to become evident. No human infection with *T*. *cruzi* via the oral route has yet been reported in the Gran Chaco region, and transmission via blood transfusion or organ transplant is highly unlikely due to full coverage of donors. Some sylvatic triatomine species may colonize peridomestic habitats and contributed to domestic transmission of *T*. *cruzi* elsewhere, as with *Triatoma eratyrusiformis* in sub-Andean Argentina [65] and *T*. *sordida*, *T*. *pseudomaculata*, and *T*. *brasiliensis* in Bahia, Brazil [66]. In our study area, however, householders’ surveillance efforts revealed the occasional invasion of domiciles by adult *T*. *sordida*, and more rarely by *Panstrongylus geniculatus* [27,67]. Their role as secondary vectors of *T*. *cruzi* to humans in Pampa del Indio appears to be marginal, if any. The local *T*. *sordida* populations (member of a species complex differing in several traits relevant to tendency toward domesticity and transmission) were tightly associated with chickens in peridomestic habitats and failed to colonize human sleeping quarters before or after interventions in Area I and III [27,67]. The baseline prevalence of *T*. *cruzi* infection in peridomestic *T*. *sordida* in Area I (2% by optic microscopy and 6% by kDNA-PCR) remained unchanged slightly after community-wide insecticide spraying [58]. No *T*. *cruzi*-infected *P*. *geniculatus* has been found so far in the few specimens collected. In both triatomine species, the degree of human-vector contact (as determined by bloodmeal analysis) was marginal. The introduction of sylvatic genotypes of *T*. *cruzi* into domestic habitats apparently occurred rarely both in the humid and dry Argentine Chaco over the last few decades (e.g., [68–72]).

Vertical transmission was the most likely source of the 8 seropositive children born after interventions in Pampa del Indio over 2010–2017, as may be the case in other hyperendemic settings under sustained, effective triatomine control actions. The observed fraction of putative congenital cases born to *T*. *cruzi*-seropositive women after interventions (3.8%) was quite close to the expected average of 4.7% [73]. None of them were detected by routine procedures, suggesting that only a small fraction of all congenital cases is regularly notified to the surveillance system and treated in Argentina [74,75]. In recognition of the large magnitude of congenital *T*. *cruzi* transmission, Argentina joined an international program (EMTCT plus) to eliminate mother-to-child transmission of HIV, syphilis, Chagas disease, and perinatal hepatitis B [76]. A prerequisite to achieve this goal is to provide universal access to serodiagnosis of *T*. *cruzi* infection, which is hard to achieve in remote rural areas, and required several serosurveys in Pampa del Indio [36]. Our close collaboration with the local public health service provided enhanced access to diagnosis and treatment of rural residents. Most children ≤18 years of age treated with benznidazole cleared parasitemia as determined by real-time PCR [34]. Etiological treatment of women in reproductive age or earlier may further decrease vertical transmission [73].

The very low seroprevalence of *T*. *cruzi* recorded in dogs (1.5%) at a district-wide scale represents a massive decrease from 26.0% recorded in Area I in 2008 slightly after program onset [24]. As an external comparison group, dog seroprevalence was 14.8% (32 of 217) in high-risk houses mainly around Pampa del Indio municipality in 2013 [77]. One-third of the few *T*. *cruzi*-seropositive dogs found a decade after program onset had been born before initial interventions and survived well beyond the mean life expectancy. The occurrence of infected dogs born after interventions was marginal as it occurred in <1% of all rural houses. Household risk stratification readily captured the increased prevalence of *T*. *cruzi* infection of dogs residing in houses ever found infested during the surveillance phase, supporting their use as sentinels of domestic transmission. Selective insecticide spraying of these houses ultimately suppressed the incipient foci but the time lag between house reinfestation, detection and selective spraying may have opened a window of opportunity for transmission to dogs. Focal vector-borne transmission to dogs may have occurred over the initial years post-intervention in three houses with transient infestation, but vertical transmission cannot be ruled out except in the incident case, which seroconverted between 2013 and 2016. Transient house infestation over the surveillance phase was also accompanied by incident canine cases elsewhere in the Argentine Chaco [14]. Lack of information on maternal serostatus of the infected dogs (S1 Table) implies that the underlying transmission route remains undefined.

The force of infection was higher in dogs than in humans, likely reflecting additional sources of infection in dogs. For example, vector-borne transmission in peridomestic habitats may explain why canine cases preceded human cases [14]. Moreover, dog infection was unrelated to the individual’s origin (urban, rural immigrant and native), probably reflecting other background transmission risks (vertical, oral, sylvatic) in unrestrained dogs [78]. Although the role of carnivory as a transmission route has been questioned [79], armadillos were a locally abundant, highly infectious reservoir host of *T*. *cruzi* [68] and a typical target prey for subsistence hunters in the Gran Chaco. Dogs often had access to, or were deliberately fed with, blood and viscera of recently killed wild mammals. Although dogs may also acquire the infection through ingestion of sylvatic or domestic triatomines [80], the likelihood of such event in Pampa del Indio appeared to be marginal after interventions.

Our results and derived inferences have some limitations. First, our study lacks the evidence-based power of a randomized controlled trial as the before-after intervention design lacked control communities. Other relevant changes may have occurred in parallel, which in theory may enhance or diminish the impact of interventions [81], or even be ineffectual. However, it is well established that in the absence of effective control actions domestic infestation and *T*. *cruzi* transmission persist in the Gran Chaco, and leaving untreated control communities is ethically unacceptable. Second, the risk-stratified sampling design was mainly based on the recorded presence of *T*. *infestans* in identified rural houses as determined by timed-manual searches and householders’ bug notifications; their joint use increased the chances of detecting house infestation (e.g., [27]). Third, house location and occupancy status did not always remain fixed across the follow-up depending on household ethnic background and specific area. In Area III, household mobility modified the positive association between domiciliary infestation and the relative odds of a child being seropositive to *T*. *cruzi*: children from mover households were at a lower risk than non-movers after allowing for house infestation status [36]. Movers were difficult to relocate and less likely to be included in the study sample [27]. Fourth, since older adult people frequently participate less often in serosurveys [31], their seroprevalence was likely underestimated both in tested residents and in those who self-reported their serostatus. Fifth, the expected imprecision in the reported ages of dogs adds undesirable variability to force-of-infection estimates. In addition, when seroprevalence is low as in our post-intervention scenario, serological tests are expected to have a lower positive predictive value than before interventions. Using two high-performance tests for screening survey purposes (plus additional testing in case of serological discordance) minimized the chance of spurious results, and we only recorded 11 discordant results. If specificity would have been an issue in our study, it did not reflect in the seroreactivity outcomes of tested children born after interventions, and the intervention would have achieved even larger impacts than the observed ones. Lastly, some land-use changes occurred in parallel to interventions. Nearly 40% of the landscape shifted from forest fragments to scrub or barren soil over 2007–2015 in Area I [82], but the pace and extent of land-use change in other operational areas may have been quite different and occurred long before program onset. Although in theory such land-use changes may affect the population dynamics of sylvatic *T*. *sordida* and other triatomines (including their dispersal rates), its direct effects on domestic transmission mediated by *T*. *infestans* would at best be tenuous in the presence of sustained vector control actions leading to rare house infestation.

On the strengths side, our study used risk-stratified sampling design to increase the chances of detecting new human or canine cases, rather than simple random sampling or convenience sampling; the former may be statistically inefficient and the latter prone to severe bias. We used the same serological tests and procedures throughout the follow-up; included two tests to minimize the likelihood of false negative and false positive results (including cross-reactivity with other trypanosomes or *Leishmania* sp. [83] in dogs), and additional testing to untie serologically discordant outcomes. Xenodiagnosis confirmed several of the native canine cases born after program onset. Relocating people (and dogs) in disperse rural areas was challenging and probably explains the paucity of direct estimates of the incidence of human or dog infection (e.g., [14,16,62,84]). The georeferenced database allowed us to match house infestation data with dog and human infection status; link child to maternal serostatus and vector exposure, and assess transmission status at a district-wide scale. Estimating the force of infection both retrospectively and prospectively provided robust evidence of its large decrease.

The outcomes of this long-term intervention program are pertinent to a broader endemic region beyond Pampa del Indio whose exact limits are hard to define. The Gran Chaco region is spatially heterogeneous in terms of vector-borne transmission and the distribution of socio-demographic determinants relevant to Chagas disease, thus creating a disease mosaic. Accelerated rural development in some of its sections over the last decades indirectly reduced the risk of vector-borne transmission, but this cannot be generalized and it certainly does not apply to most of Chaco Province and Pampa del Indio in particular.

The domestic transmission of *T*. *cruzi* mediated by *T*. *infestans* in the Gran Chaco is highly resilient. Whenever community-wide house spraying with insecticide is implemented without a consistent vector surveillance system, transient declines in the force of infection are followed by rapid recovery in the Argentine and Bolivian Chaco [14,85], and elsewhere [63]. Our current results, in a highly endemic area inhabited by vulnerable subgroups in heterogeneous rural communities [31], evince that interrupting vector-borne transmission of *T*. *cruzi* to humans in hyperendemic areas is feasible with the current means implemented systematically. Scaling up the intervention program, carefully tailored to local settings and paying due attention to the eventual emergence of pyrethroid resistance, is the next challenge.

## Supporting information

S1 Text Description of the study area. (DOCX)

S2 Text Checklist of STROBE recommendations for observational studies. (DOC)

S1 Fig Flow diagram of the study design. (PDF)

S2 Fig Flow chart of the study houses. (PDF)

S1 Table Risk factors for *T*. *cruzi*-seropositive, native dogs born after interventions with permanent residence in the study area, Pampa del Indio, 2016. (DOCX)

S2 Table Individual-level human and dog database. (XLSX)

## References

[1] Global Health Metrics. https://www.thelancet.com/pb-assets/Lancet/gbd/summaries/diseases/chagas-disease.pdf

[2] World Health Organization. Research priorities for Chagas disease, human African trypanosomiasis and leishmaniasis. 2018; Technical report series; no. 975. 23484340

[3] Perez JC, Lymbery AL, Thompson RCA. Reactivation of Chagas Disease: implications for global health. Trends in Parasitol. 2015;31(11):595–603. 10.1016/j.pt.2015.06.006 26458782

[4] World Health Organization. Chagas disease in Latin America: an epidemiological update based on 2010 estimates. Wkly Epidemiol Rec. 2015;33–44. 25671846

[5] World Health Organization. World Health Statistics Overview: Monitoring Health for the SDGs, Sustainable Development Goals. Geneva. 2019 Licence: CC BY-NC-SA 3.0 IGO https://apps.who.int/iris/bitstream/handle/10665/311696/WHO-DAD-2019.1-eng.pdf?ua=1

[6] Gürtler RE, Cecere MC. Chagas disease vector control. In: Triatominae: The Biology of Chagas Disease Vectors. Guarneri AA, Lorenzo MG (eds). Springer. 2021;New York, 291 pag.

[7] Silveira, AC. El control de la enfermedad de Chagas en los países del Cono Sur de América. Historia de una iniciativa internacional 1991/2001 Silveira AC (Ed.). PAHO, Argentina. 2002.

[8] World Health Organization. Keeping the vector out: housing improvements for vector control and sustainable development. 2017; World Health Organization, Geneva. Licence: CC BY-NC-SA 3.0 IGO.

[9] Panamerican Health Organization, 2018. https://www.paho.org/par/index.php?option=com_content&view=article&id=2011:mision-internacional-certifica-corte-de-transmision-vectorial-de-chagas-en-paraguay&Itemid=258

[10] National Ministry of Health, 2018. https://www.argentina.gob.ar/noticias/argentina-recibio-reconocimiento-internacional-por-certificar-la-eliminacion-de-la (accessed on January 19th, 2021).

[11] Gürtler RE, Cardinal MV. Reservoir host competence and the role of domestic and commensal hosts in the transmission of *Trypanosoma cruzi*. Acta Trop. 2005;151, 32–50. 10.1016/j.actatropica.2015.05.029 26051910

[12] Cohen JE, Gürtler RE. Modeling household transmission of American trypanosomiasis. Science. 2001;293:694–8 10.1126/science.1060638 11474111

[13] Cardinal MV, Sartor PA, Gaspe MS, Gürtler RE. High levels of human infection with *Trypanosoma cruzi* associated with the domestic density of infected vectors and hosts in a rural area of northeastern Argentina. Parasites & Vectors.2018;11:492. 10.1186/s13071-018-3069-0 30165892 PMC6118006

[14] Gürtler RE, Kitron U, Cecere MC, Segura EL, Cohen JE. Sustainable vector control and management of Chagas disease in the Gran Chaco, Argentina. Proc Natl Acad Sci USA.2007;104(41):16194–16199. 10.1073/pnas.0700863104 17913895 PMC2042184

[15] Arce-Fonseca M, Carrillo-Sánchez SC, Molina-Barrios RM, Martínez-Cruz M, Cedillo-Cobián JR, Henao-Díaz YA, et al. Seropositivity for *Trypanosoma cruzi* in domestic dogs from Sonora, Mexico. Infect Dis Poverty.2017;6(1):120. 10.1186/s40249-017-0333-z 28870247 PMC5584529

[16] Cardinal MV, Castañera MB, Lauricella MA, Cecere MC, Ceballos LA, Vazquez-Prokopec GM, et al. A prospective study of the effects of sustained vector surveillance following community-wide insecticide application on *Trypanosoma cruzi* infection of dogs and cats in rural northwestern Argentina. Am J Trop Med Hyg.2006;75:753–761. 17038707 PMC1853286

[17] Cardinal MV, Lauricella MA, Marcet PL, Orozco MM, Kitron U, Gürtler RE. Impact of community-based vector control on house infestation and *Trypanosoma cruzi* infection in *Triatoma infestans*, dogs and cats in the Argentine Chaco. Acta Tropica.2007;103:201–211. 10.1016/j.actatropica.2007.06.007 17686448 PMC2931801

[18] Castillo-Neyra R, Chou Chu L, Quispe-Machaca V, Ancca-Juarez J, Malaga Chavez FS, Bastos Mazuelos M, Naquira C, et al. The potential of canine sentinels for reemerging *Trypanosoma cruzi* transmission. Prev Vet Med. 2015;120(3–4):349–56. 10.1016/j.prevetmed.2015.04.014 25962956 PMC4657134

[19] Freitas YBN, Souza CDSF, Magalhães JME, Sousa MLR, d’Escoffier LN, Valle TZD, et al. Natural infection by *Trypanosoma cruzi* in triatomines and seropositivity for Chagas disease of dogs in rural areas of Rio Grande do Norte, Brazil. Rev Soc Bras Med Trop.2018;51(2):190–197. 10.1590/0037-8682-0088-2017 29768552

[20] Jaimes-Dueñez J, Triana-Chávez O, Cantillo-Barraza O, Hernández C, Ramírez JD, Góngora-Orjuela A. Molecular and serological detection of *Trypanosoma cruzi* in dogs (*Canis lupus familiaris*) suggests potential transmission risk in areas of recent acute Chagas disease outbreaks in Colombia. Prev Vet Med.2017;6 1;141:1–6. 10.1016/j.prevetmed.2017.03.009 Epub 2017 Mar 31. 28532988

[21] Tenney TD, Curtis-Robles R, Snowden KF, Hamer SA. Shelter dogs as sentinels for *Trypanosoma cruzi* transmission across Texas. Emerg Infect Dis.2014;20(8):1323–6. 10.3201/eid2008.131843 25062281 PMC4111162

[22] Roque ALR, Jansen AM. The importance of sentinel domestic animals to identify risk areas to the emergence of Chagas disease. Rev Soc Bras Med Trop 2008;41:(Supl III) 191–193.

[23] Gabrielli S, Spinicci M, Macchioni F, Rojo D, Totino V, Rojas P et al. Canine *Trypanosoma cruzi* infection in the Bolivian Chaco. Parasites Vectors 2018:11:632. 10.1186/s13071-018-3247-0 30541629 PMC6292100

[24] Cardinal MV, Orozco MM, Enriquez GF, Ceballos LA, Gaspe MS, Alvarado-Otegui JA, et al. Heterogeneities in the eco-epidemiology of *Trypanosoma cruzi* infection in rural communities of the Argentinean Chaco. Am J Trop Med Hyg. 2014;90:1063–1073. 10.4269/ajtmh.13-0251 24732461 PMC4047730

[25] Roque ALR, Xavier SCC, Gerhardt M, Silva MFO, Lima VS, D’Andrea PS et al. *Trypanosoma cruzi* among wild and domestic mammals in different areas of the Abaetetuba municipality (Pará State, Brazil), an endemic Chagas disease transmission area. Vet Parasitol 2013;193:71–77. 10.1016/j.vetpar.2012.11.028 23261089

[26] Gaspe M, Provecho Y, Cardinal M, Fernández M, Gürtler RE. Ecological and sociodemographic determinants of house infestation by *Triatoma infestans* in indigenous communities of the Argentine Chaco. PLoS Negl Trop Dis.2015;9:e0003614. 10.1371/journal.pntd.0003614 25785439 PMC4364707

[27] Gaspe MS, Provecho YM, Fernández MP, Vassena CV, Santo Orihuela PL, Gürtler RE. Beating the odds: sustained Chagas disease vector control in remote indigenous communities of the Argentine Chaco over a seven-year period. PLoS Negl Trop Dis.2018;12(10):e0006804. 10.1371/journal.pntd.0006804 30278044 PMC6168123

[28] Gurevitz JM, Ceballos LA, Gaspe MS, Alvarado-Otegui JA, Enríquez GF, Kitron U, et al. Factors affecting infestation by *Triatoma infestans* in a rural area of the humid Chaco in Argentina: a multi-model inference approach. PLoS Negl. Trop. Dis.2011;5, e1349, 10.1371/journal.pntd.0001349 22028941 PMC3196485

[29] Gurevitz JM, Gaspe MS, Enriquez GF, Provecho YM, Kitron U, Gürtler RE. Intensified surveillance and insecticide-based control of the Chagas disease vector *Triatoma infestans* in the Argentinean Chaco. PLoS Negl Trop Dis. 2013;7:e2158. 10.1371/journal.pntd.0002158 23593525 PMC3623707

[30] Provecho YM, Gaspe MS, Fernández MDP, Gürtler RE. House reinfestation with *Triatoma infestans* (Hemiptera: Reduviidae) after community-wide spraying with insecticides in the Argentine Chaco: a multifactorial process. J Med Entomol.2017;54(3):646–657. 10.1093/jme/tjw224 28399199

[31] Fernández MP, Gaspe MS, Sartor PA, Gürtler RE. Human *Trypanosoma cruzi* infection is driven by eco-social interactions in rural communities of the Argentine Chaco. PLOS Negl Trop Dis.2019a. 10.1371/journal.pntd.0007430 31841558 PMC6936860

[32] Gürtler RE, Cardinal MV, Provecho YM, Enriquez GF, Macchiaverna NP, Rodríguez Planes LI et al. Hacia la eliminación de *Triatoma infestans* en el Municipio de Pampa del Indio, Chaco: efectos de la implementación de un programa de control integrado durante una década. XVIII Simposio Internacional sobre Enfermedades Desatendidas, CABA, 5–6 october 2017. https://www.mundosano.org/wp-content/uploads/2018/03/libro-de-resumenes-2017.pdf

[33] Morello J, Matteucci SD, Rodríguez AF, Silva M. Ecorregiones y complejos ecosistémicos argentinos. 1a ed. Buenos Aires, Argentina. Orientación Gráfica Editora, 2012. 752p

[34] Gurevitz JM, Gaspe MS, Enríquez GF, Vassena CV, Alvarado-Otegui JA, Provecho YM, et al. Unexpected failures to control Chagas disease vectors with pyrethroid spraying in northern Argentina. J Med Entomol.2012;49:1379–86. 10.1603/me11157 23270166 PMC3760256

[35] Sartor P, Colaianni I, Cardinal MV, Bua J, Freilij H, Gürtler RE. Improving access to Chagas disease diagnosis and etiologic treatment in remote rural communities of the Argentine Chaco through strengthened primary health care and broad social participation. PLoS Negl Trop Dis.2017;11:e0005336. 10.1371/journal.pntd.0005336 28192425 PMC5325580

[36] Fernández MP, Gaspe MS, Gürtler RE. Inequalities in the social determinants of health and Chagas disease transmission risk in indigenous and creole households in the Argentine Chaco. Parasites Vectors.2019;12,184. 10.1186/s13071-019-3444-5 31029147 PMC6487000

[37] Macchiaverna NP, 2019. El rol de los humanos en la epidemiología molecular del ciclo doméstico de transmisión del *Trypanosoma cruzi* en un área rural del Chaco Argentino. University of Buenos Aires. PhD Thesis.

[38] World Health Organization. Anti-*Trypanosoma cruzi* assays: operational characteristics. Report 1. World Health Organization, 2010.

[39] Enriquez GF, Cardinal MV, Orozco MM, Schijman AG, Gürtler RE. Detection of *Trypanosoma cruzi* infection in naturally infected dogs and cats using serological, parasitological and molecular methods. Acta Trop.2013;126:211–17. 10.1016/j.actatropica.2013.03.001 23499860 PMC3675883

[40] Brown LD, Cai TT, DasGupta A. Interval estimation for a binomial proportion. Statistical Science.2001;16:101–17.

[41] Pérez GE, Conte A, Garde JE, Messori S, Vanderstichel R, Serpell J. Movement and home range of owned free-roaming male dogs in Puerto Natales, Chile. Applied Animal Behaviour Science.2018;205:74–82. 10.1016/j.applanim.2018.05.022

[42] R Development Core Team. R: a language and environment for statistical computing. Vienna: R Foundation for Statistical Computing; 2012.

[43] Dorai-Raj, S. binom: Binomial Confidence Intervals For Several Parameterizations. 2014. https://CRAN.R-project.org/package=binom

[44] Bates D, Mächler M, Bolker B, Walker S. Fitting Linear Mixed-Effects Models Using lme4. Journal of Statistical Software. 2015;67(1):1–48. 10.18637/jss.v067.i01

[45] Elzhov TV, Mullen KM, Spiess AN, Bolker B. Minpack.lm: R Interface to the Levenberg-Marquardt Nonlinear Least-Squares Algorithm Found in MINPACK, Plus Support for Bounds. 2016. https://CRAN.R-project.org/package=minpack.lm

[46] Abad-Franch F, Valenca-Barbosa C, Sarquis O, Lima MM. All that glisters is not gold: Sampling-process uncertainty in disease-vector surveys with false-negative and false-positive detections. PLoS Negl Trop Dis. 2014;8(9):e3187. 10.1371/journal.pntd.0003187 25233352 PMC4169387

[47] OPS—Organización Panamericana de Salud. Guía de evaluación de los procesos de control de triatominos y del control de la transmisión transfusional de *T. cruzi*, OPS/HCP/HCT/196.02, OPS, Washington, 2002;8 pp

[48] Ostermayer AL, Passos ADC, Silveira AC, Ferreira AW, Macedo V, Prata AR. O inquérito nacional de soroprevalência de avaliação do controle da doença de Chagas no Brasil (2001–2008). Rev Soc Bras Med Trop. 2011;44 (Suppl. 2):S108–S121. 10.1590/s0037-86822011000800015 21584364

[49] Russomando G, Cousiño B, Sanchez Z, Franco LX, Nara EM, Chena L et al. Chagas disease: national survey of seroprevalence in children under five years of age conducted in 2008. Mem. Inst. Oswaldo Cruz. 2017;112:348–353. 10.1590/0074-02760160407 28443980 PMC5398161

[50] Lorca HM, García CA, Bahamonde MMI, Fritz MA, Tassara OR. Serological certification of the interruption of the vectorial transmission of Chagas disease in Chile Rev. méd. Chile. 2001;129:264–69. 11372293

[51] Abad-Franch F, Diotaiuti L, Gurgel-Gonçalvez R, Gürtler RE. On bugs and bias: improving Chagas disease control assessment. Mem Inst Oswaldo Cruz. 2014;109:125–130. 24809110

[52] Collaborating Group on Chagas Disease Modelling. Insights from quantitative and mathematical modelling on the proposed WHO 2030 goals for Chagas disease [version 1; peer review: 2 approved] Gates Open Research 2019:1539. (10.12688/gatesopenres.13069.1) PMC6856696 31781687

[53] Gürtler RE, Fernández MP, Cardinal MV. Eco-epidemiology of the domestic vector-borne transmission of *Trypanosoma cruzi*. In: Triatominae: The Biology of Chagas Disease Vectors. Guarneri AA, Lorenzo MG (eds). Springer. New York, 2021;291 pag.

[54] Cucunubá ZM, Nouvellet P, Peterson JK, Bartsch SM, Lee BY, Dobson AP, et al. Complementary paths to Chagas Disease elimination: the impact of combining vector control with etiological treatment. Clin Infect Dis. 2018;66(suppl_4):S293–S300. 10.1093/cid/ciy006 29860294 PMC5982731

[55] Cecere MC, Castañera MB, Canale DM, Chuit R, Gürtler RE. *Trypanosoma cruzi* infection in *Triatoma infestans* and other triatomines: long-term effects of a control program in a rural area of northwestern Argentina. Pan American Journal of Public Health, 1999;5:392–399. 10.1590/s1020-49891999000500003 10446505

[56] Marcet PL, Duffy T, Cardinal MV, Burgos JM, Lauricella MA, Levin MJ et al. PCR-based identification of *Trypanosoma cruzi* lineages in feces of triatomine bugs from rural northwestern Argentina. Parasitology, 2006;132:57–65. 10.1017/S0031182005008772 16393354 PMC1853270

[57] Ceballos LA, Piccinali RV, Marcet PL, Vazquez-Prokopec GM, Cardinal MV, Schachter-Broide J et al. Hidden sylvatic foci of the main vector of Chagas disease *Triatoma infestans*: threats to the vector elimination campaign? PLoS Neglected Tropical Diseases, 2011;5:e1349. 22039559 10.1371/journal.pntd.0001365 PMC3201917

[58] Macchiaverna NP, Gaspe MS; Enriquez GF, Tomassone L, Gürtler RE, Cardinal MV. *Trypanosoma cruzi* infection in *Triatoma sordida* before and after community-wide residual insecticide spraying in the Argentinean Chaco. Acta Tropica. 2015;143, 97–102. 10.1016/j.actatropica.2014.12.010 25579426

[59] Alvarado-Otegui JA, Ceballos LA, Orozco MM, Enríquez G, Cardinal MV, Schijman AG, et al. The sylvatic transmission cycle of *Trypanosoma cruzi* in the humid Chaco of Argentina. Acta Tropica, 2012;124:79–86. 10.1016/j.actatropica.2012.06.010 22771688 PMC3444808

[60] Ceballos LA, Piccinali RV, Berkunsky I, Kitron U, Gürtler RE. First finding of melanic sylvatic *Triatoma infestans* (Hemiptera: Reduviidae) in the Argentine Chaco. J Med Entomol, 2009;46(5):1195–1202. 10.1603/033.046.0530 19769054 PMC2782367

[61] Macchiaverna NP, Enriquez GF, Bua J, Fernández MP, Sartor PA, Gürtler RE, et al. Human infectiousness and parasite load in chronic patients seropositive for *Trypanosoma cruzi* in a rural area of the Argentine Chaco. Infect Genet Evol. 2020;78:104062. 10.1016/j.meegid.2019.104062 31683004

[62] Mott KE, Munis TC, Lehman J, Hoff R, Morrow RH, de Oliveira TS, et al. House construction, triatomine distribution, and household distribution of seroreactivity to *Trypanosoma cruzi* in a rural community in Northeastern Brazil. Am J Trop Med Hyg. 1978;27:1116–1122. 10.4269/ajtmh.1978.27.1116 103445

[63] Piesman J, Sherlock IA, Mota E, Todd CW, Hoff R, Weller TH. Association between household triatomine density and incidence of *Trypanosoma cruzi* infection during a nine-year study in Castro Alves, Bahia, Brazil. Am J Trop Med Hyg. 1985;34:866–869. 10.4269/ajtmh.1985.34.866 3929635

[64] Delgado S, Castillo Neyra R, Quispe Machaca VR, Ancca Juárez J, Chou Chu L, et al. A history of Chagas Disease transmission, control, and re-emergence in peri-rural La Joya, Peru. PLoS Negl Trop Dis. 2011;5(2):e970. 10.1371/journal.pntd.0000970 21364970 PMC3042997

[65] Cecere MC, Leporace M, Fernández MP, Zárate JE, Moreno C, Gürtler RE. Host-feeding sources and infection by *Trypanosoma cruzi* of *Triatoma infestans* and *Triatoma eratyrusiformis* (Hemiptera, Reduviidae) from the Calchaqui Valleys in Northwestern Argentina. Journal of Medical Entomology. 2016;55:666–673 10.1093/jme/tjw002 26849898

[66] Ribeiro G, dos Santos CGS, Lanza F, Reis J, Vaccarezza F, Diniz C, et al. Wide distribution of *Trypanosoma cruzi*-infected triatomines in the State of Bahia, Brazil. Parasites Vectors. 2019;12, 604. 10.1186/s13071-019-3849-1 31878960 PMC6933904

[67] Rodríguez-Planes LI, Gaspe MS, Enriquez GF, Gürtler RE. Impacts of residual insecticide spraying on house infestation with *Triatoma sordida* and co-occurrence of *Triatoma infestans*: a three-year follow-up in northeastern Argentina. Acta Tropica, 202:105251, 2020. 10.1016/j.actatropica.2019.105251 31706862

[68] Orozco MM, Enriquez GF, Alvarado-Otegui JA, Cardinal MV, Schijman AG, Kitron U, et al. New sylvatic hosts of *Trypanosoma cruzi* and their reservoir competence in the humid Chaco of Argentina: a longitudinal study. Am J Trop Med Hyg. 2013;88:872–882. 10.4269/ajtmh.12-0519 23530075 PMC3752751

[69] Enriquez GF, Cardinal MV, Orozco MM, Lanatti L, Schijman AG, Gürtler RE. Discrete typing units of *Trypanosoma cruzi* identified in rural dogs and cats in the humid Argentinean Chaco. Parasitology, 2012;140(3):303–308. 10.1017/S003118201200159X 23058180 PMC3721149

[70] Macchiaverna NP, Enriquez GF, Buscaglia CA, Balouz V, Gürtler RE, Cardinal MV. New human isolates of *Trypanosoma cruzi* confirm the predominance of hybrid lineages in domestic transmission cycles of the Argentinean Chaco. Infect Genetics Evol, 2018;66:229–235. 10.1016/j.meegid.2018.10.001 30296602

[71] Cardinal MV, Lauricella MA, Ceballos LA, Lanati L, Marcet PL, Levin JM et al. Molecular epidemiology of domestic and sylvatic *Trypanosoma cruzi* infection in rural northwestern Argentina. Int J Parasitol, 2008;38(13):1533–1543. 10.1016/j.ijpara.2008.04.010 18585717 PMC3143243

[72] Diosque P, Barnabé C, Padilla A, Marco J, Cardozo R, Cimino R, et al. Multilocus enzyme electrophoresis analysis of *Trypanosoma cruzi* isolates from a geographically restricted endemic area for Chagas’ disease in Argentina. Int. J. Parasitol, 2003;33:997–1003. 10.1016/s0020-7519(03)00139-5 13129520

[73] Carlier Y, Altcheh J, Angheben A, Freilij H, Luquetti AO, Schijman AG, et al. Congenital Chagas disease: updated recommendations for prevention, diagnosis, treatment, and follow-up of newborns and siblings, girls, women of childbearing age, and pregnant women. PLoS Negl Trop Dis. 2019;13:e0007694. 10.1371/journal.pntd.0007694 31647811 PMC6812740

[74] Gürtler RE, Segura EL, Cohen JE. Congenital transmission of *Trypanosoma cruzi* infection in Argentina. Emerging Infectious Diseases. 2003 9(1):29. 10.3201/eid0901.020274 12533278 PMC2873757

[75] Danesi E, Codebó MO, Sosa-Estani S. Transmisión congénita de *Trypanosoma cruzi*. Argentina 2002–2014. Medicina (Buenos Aires). 2019;79(2):81–89. 31048272

[76] Panamerican Health Organization. EMTCT Plus: Framework for elimination of mother-to-child transmission of HIV, Syphilis, Hepatitis B, and Chagas. 2017. https://iris.paho.org/handle/10665.2/34306

[77] Enriquez GF, Garbossa G, Macchiaverna NP, Argibay HD, Bua J, Gürtler RE. Is the infectiousness of dogs naturally infected with *Trypanosoma cruzi* associated with poliparasitism? Veterinary Parasitology. 2016;223:186–194. 10.1016/j.vetpar.2016.04.042 27198799

[78] Barr SC. Canine Chagas’ disease (American trypanosomiasis) in North America. Vet Clin North Am Small Anim Pract. 2009;39:1055–1064. 10.1016/j.cvsm.2009.06.004 19932362

[79] Roellig DM, Ellis AE, Yabsley MJ. Oral transmission of *Trypanosoma cruzi* with opposing evidence for the theory of carnivory. J Parasitol. 2009;95:360–4. 10.1645/GE-1740.1 18763853 PMC2911628

[80] Montenegro V, Jiménez M, Pinto Dias J, Zeledón R. Chagas disease in dogs from endemic areas of Costa Rica. Mem Inst Oswaldo Cruz. 2002;97:491–494. 10.1590/s0074-02762002000400006 12118277

[81] Wilson AL, Boelaert M, Kleinschmidt I, Pinder M, Scott TW, Tusting LS, et al. Evidence-based vector control? Improving the quality of vector control trials. Trends in Parasitology, 2015;31 (8):380–390. 10.1016/j.pt.2015.04.015 25999026

[82] Rodriguez-Planes LI, 2018. Análisis espacio-temporal de los patrones de infestación por vectores de la Enfermedad de Chagas en viviendas rurales del Chaco argentino: domesticación y heterogeneidad. University of Buenos Aires. PhD Thesis.

[83] Gürtler RE, Cardinal MV. Dogs and their role in the eco-epidemiology of Chagas disease. In: Dog Parasites Endangering Human Health. Strube Christina & Mehlhorn Heinz Eds. Parasitology Research Monograph 13. Springer. 2020. ISBN 978-3-030-53229-1 13.

[84] Newton-Sánchez OA, Espinoza-Gómez F, Melnikov V, Delgado-Enciso I, Rojas-Larios F, Dumonteil E. Seroprevalence of *Trypanosoma cruzi* (TC) and risk factors in Colima, Mexico. Gac Med Mex. 2017;153:179–184 28474704

[85] Samuels AM, Clark EH, Galdos-Cardenas G, Wiegand RE, Ferrufino L, et al. Epidemiology of and impact of insecticide spraying on Chagas Disease in communities in the Bolivian Chaco. PLoS Negl Trop Dis. 2013;7(8):e2358. 10.1371/journal.pntd.0002358 23936581 PMC3731239

