# Supplementary material for: Long-term impact of a ten-year intervention program on human and canine Trypanosoma cruzi infection in the Argentine Chaco
Source: PLoS Negl Trop Dis. 2021 May 12;15(5):e0009389. doi: 10.1371/journal.pntd.0009389 (PMC8115854; doi:10.1371/journal.pntd.0009389)
Supplement: S1 Text — (DOCX) [file pntd.0009389.s001.docx]

S1 Text. Brief description of the study area.

**Pampa del Indio** municipality is situated exactly in the transition between the dry (west) and humid (eastern) Argentine Chaco. The climate is continental, warm, with rainfall occurring mainly in summer. Annual mean temperature is 23°C (mean annual minimum and maximum temperatures, 17 and 29°C, respectively). Average annual rainfall historically has been 954 mm. The landscape is flat and land use ranges from humid forest developed near riverbanks, native dry forest (preserved in the Provincial Park) to crop fields, resulting in a mosaic of patches with various degrees of degradation [1]. Main crops include cotton, corn, pumpkin, watermelon, melon, and more recently, soybean. Rural residents live mostly on a subsistence economy, cultivating or raising livestock (mainly goats, but also cows and occasionally sheep). The area is inhabited by two main ethnic groups: creoles of European descent and qom, which originally were a nomadic indigenous people.

**Area I** encompassed 327 inhabited house compounds in an area of 450 km^2^ as of 2007. There were 26

uninhabited or abandoned houses and 37 public buildings (including 11 schools and 5 primary health care centers). Qom people represented 24% of the 1,187 inhabitants of Area I and occupied 16% of the houses. At baseline, house infestation with *T. infestans* reached 39.8% of the 327 inspected households [2]. Community-wide insecticide spraying was performed in late 2007 and house reinfestation was closely monitored. We detected high-levels of house infestation few months after spraying at least partially associated with unsuspected pyrethroid resistant populations, which were subsequently sprayed with malathion and suppressed [3,4].

**Area II** encompassed 437 inhabited house compounds, four primary schools and one secondary school, and three primary health-care centers in an area of 300 km^2^ as of 2008. Qom people comprised the majority of Area II population (67%). At baseline survey conducted in 2008, *T. infestans* was found in 20.0% of the 185 inhabited houses selected systematically and inspected for house infestation [5]. Community-wide insecticide spraying was performed in late 2008 and house reinfestation was monitored regularly. The reinfested houses were focally sprayed and a few persistent infestations associated with pyrethroid-resistant populations were sprayed with malathion. House infestation with *T. infestans* apparently decreased to nil in 2014 [5,6].

**Area III** was the most densely populated area, encompassing 407 inhabited households in 95 km^2^ as of 2008. There were 17 public buildings (4 primary schools, 3 health care centers, 6 churches and 6 community centers). The majority (90%) of the population was of qom descent, and had a growing population age structure skewed towards young age groups [7]. At baseline, in 2008, 28% of the 386 inhabited households were infested with *T. infestans.* After community-wide insecticide spraying, house infestation remained below 1%, with no infested house at 2015 [8,9].

**Area IV** comprised 257 inhabited households in 462 km^2^, including 4 primary schools, 2 health-care centers and 4 churches as of 2009. It was surveyed and sprayed with pyrethroid insecticide in two steps. A baseline household and vector survey followed by community-wide insecticide spraying took place in late 2009, encompassing 164 (66%) households. *Triatoma infestans* was found in 17.7% of them. In 2010, all 257 houses were searched for triatomines and sprayed with insecticide. House infestation fell to 7% in 2010 and remained below 10% thereafter [6].

References

1. Morello J, Matteucci SD, Rodríguez AF, Silva M. Ecorregiones y complejos ecosistémicos argentinos. 1a ed. Buenos Aires, Argentina. Orientación Gráfica Editora, 2012. 752p.
2. Gurevitz JM, Ceballos LA, Gaspe MS, Alvarado-Otegui JA, Enríquez GF, Kitron U, et al. Factors affecting infestation by *Triatoma infestans* in a rural area of the humid Chaco in Argentina: a multi-model inference approach. PLoS Negl. Trop. Dis.2011;5, e1349, <http://dx.doi.org/10.1371/journal.pntd.0001349>.
3. Gurevitz JM, Gaspe MS, Enriquez GF, Provecho YM, Kitron U, Gürtler RE. Intensified surveillance and insecticide-based control of the Chagas disease vector *Triatoma infestans* in the Argentinean Chaco. PLoS Negl Trop Dis. 2013;7:e2158.
4. Gurevitz JM, Gaspe MS, Enríquez GF, Vassena CV, Alvarado-Otegui JA, Provecho YM, et al. Unexpected failures to control Chagas disease vectors with pyrethroid spraying in northern Argentina. J Med Entomol.2012;49:1379–86.
5. Provecho YM, Gaspe MS, Fernández MDP, Gürtler RE. House reinfestation with *Triatoma infestans* (Hemiptera: Reduviidae) after community-wide spraying with insecticides in the Argentine Chaco: a multifactorial process. J Med Entomol.2017; 54(3):646-657. doi: 10.1093/jme/tjw224
6. Macchiaverna NP, 2019. El rol de los humanos en la epidemiología molecular del ciclo doméstico de transmisión del *Trypanosoma cruzi* en un área rural del Chaco Argentino. University of Buenos Aires. PhD Thesis.
7. Fernández MP, Gaspe MS, Gürtler RE. Inequalities in the social determinants of health and Chagas disease transmission risk in indigenous and creole households in the Argentine Chaco. Parasites Vectors.2019;12,184. <https://doi.org/10.1186/s13071-019-3444-5>.
8. Gaspe M, Provecho Y, Cardinal M, Fernández M, Gürtler RE. Ecological and sociodemographic determinants of house infestation by *Triatoma infestans* in indigenous communities of the Argentine Chaco. PLoS Negl Trop Dis.2015;9:e0003614.
9. Gaspe MS, Provecho YM, Fernández MP, Vassena CV, Santo Orihuela PL, Gürtler RE. Beating the odds: sustained Chagas disease vector control in remote indigenous communities of the Argentine Chaco over a seven-year period. PLoS Negl Trop Dis.2018; 12(10):e0006804. doi: 10.1371/journal.pntd.0006804.
