# Supplementary figures and images for: Long-term impact of a ten-year intervention program on human and canine Trypanosoma cruzi infection in the Argentine Chaco
Source: PLoS Negl Trop Dis. 2021 May 12;15(5):e0009389. doi: 10.1371/journal.pntd.0009389 (PMC8115854; doi:10.1371/journal.pntd.0009389)

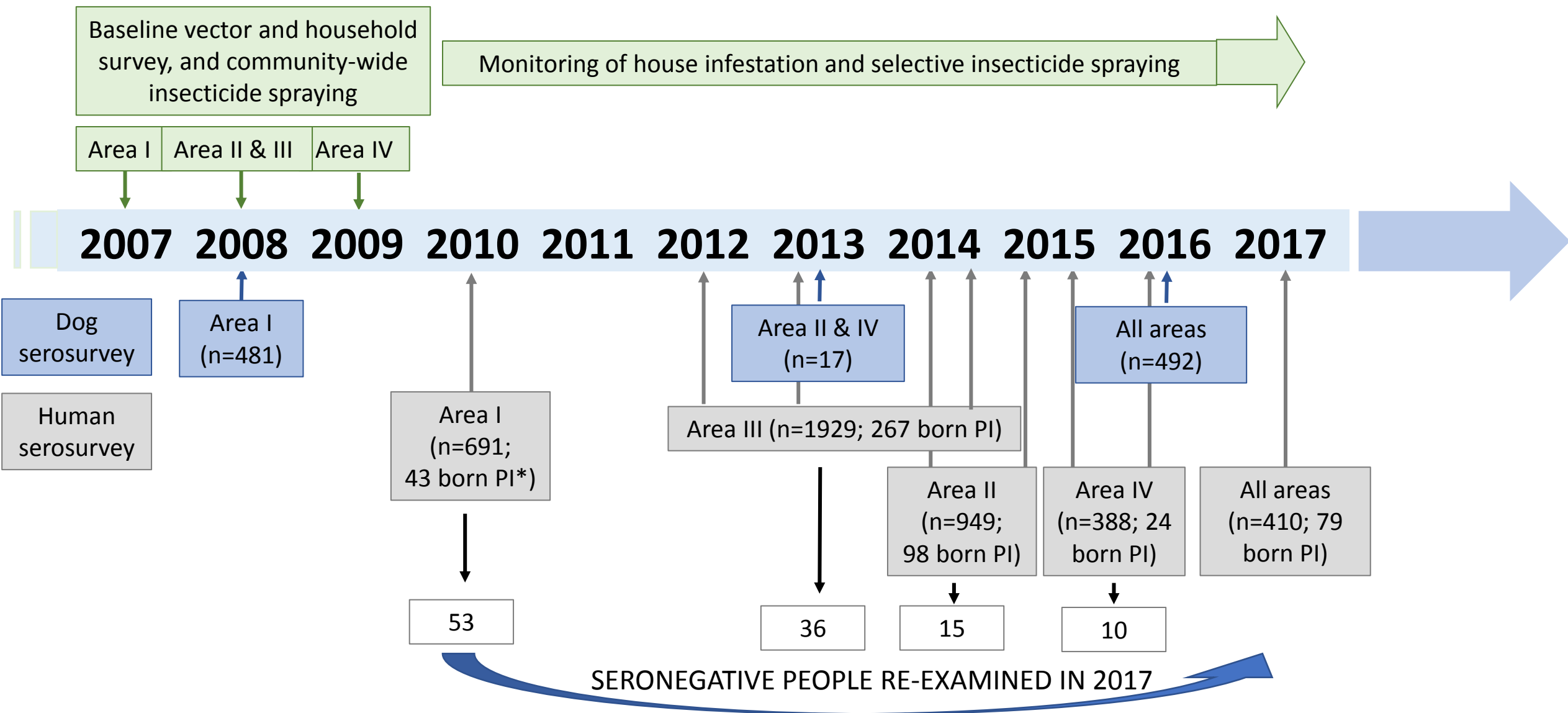

\*PI: post-interventions

Supplement: S1 Fig — (PDF) [file pntd.0009389.s003.pdf]
