# Supplementary material for: Long-term impact of a ten-year intervention program on human and canine Trypanosoma cruzi infection in the Argentine Chaco
Source: PLoS Negl Trop Dis. 2021 May 12;15(5):e0009389. doi: 10.1371/journal.pntd.0009389 (PMC8115854; doi:10.1371/journal.pntd.0009389)
Supplement: S2 Fig — (PDF) [file pntd.0009389.s004.pdf]

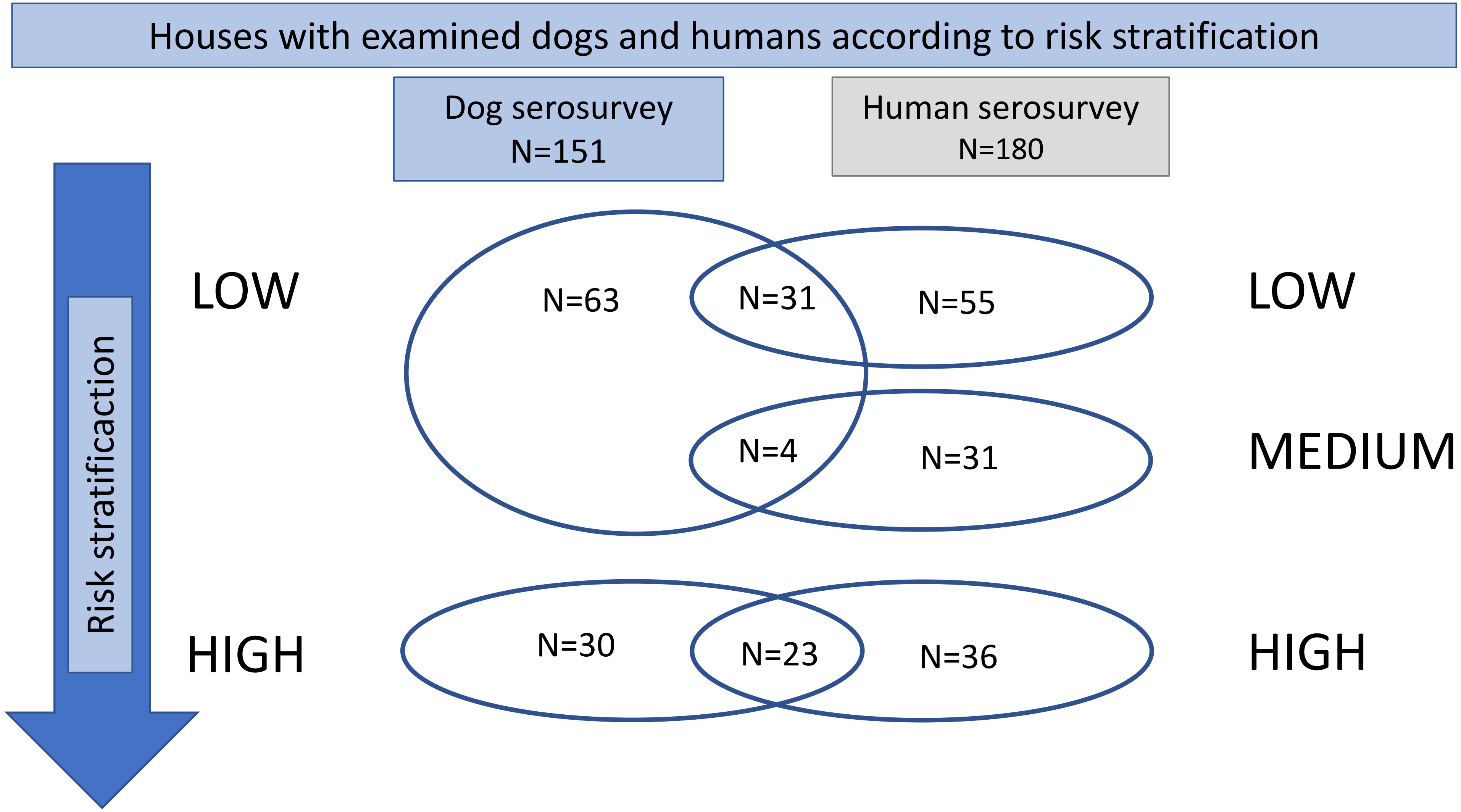
Houses with examined dogs and humans according to risk stratification
Dog serosurvey
N=151
Human serosurvey
N=180
Risk stratificacion
LOW
HIGH
N=63
N=31
N=55
N=4
N=31
N=30
N=23
N=36
LOW
MEDIUM
HIGH
