# Supplementary material for: Long-term impact of a ten-year intervention program on human and canine Trypanosoma cruzi infection in the Argentine Chaco
Source: PLoS Negl Trop Dis. 2021 May 12;15(5):e0009389. doi: 10.1371/journal.pntd.0009389 (PMC8115854; doi:10.1371/journal.pntd.0009389)
Supplement: S1 Table — (DOCX) [file pntd.0009389.s005.docx]

S1 Table. Risk factors for *T. cruzi*-seropositive, native dogs born after interventions with permanent residence in the study area, Pampa del Indio, 2016.

| Dog ID | P-12 | P-37 | P-67 | P-224 | P-466 | P-506 |
| --- | --- | --- | --- | --- | --- | --- |
| House ID | LL32 | CT8 | FB19 | LC71 | RN3 | NU31 |
| Age (years) | 3 | 8 | 6 | 5 | 7 | 5 |
| House risk | High | High | Low | Low | Low | High |
| *T. infestans* infestation date at the dog’s house during surveillance | 2016 | 2012 / 2010 / 2009 | Not infested | Not infested | Not infested | 2012 |
| Baseline *T. infestans* infestation at the dog’s house | Yes | Yes | Yes | No | No | Yes |
| Lifetime exposure to *T. infestans* | Yes | Yes | No | No | No | Yes |
| Infected bugs detected at the dog’s house (Year) | No | Yes (2009) | NA | NA | NA | No |
| No. of inhabited houses within 1 km | 1 | 7 | 6 | 40 | 12 | 4 |
| Exposure to *T. infestans* within 1 km (no. of infested houses) | No | Yes (1) | No | Yes (1) | No | No |
| Exposure to *T. sordida* at the dog’s house or within 1 km (no. of infested houses) | No | Yes (4) | Yes (2) | No | Yes (2) | No |
| Maternal serostatus for *T. cruzi* | Nd | Nd | Nd | Neg | Nd | Nd |
| Function | Guardian | Guardian | Hunter | Hunter | Hunter | Hunter |
| Number of cohabiting dogs (infected/ non-infected) | 1/3 | 0/1 | 1/3 | 0/6 | 0/6 | 0/7 |
| Number of cohabiting infected people | Nd | 1 | 1 | Nd | 2 | 5 |
| Serostatus in 2013 | Nd | Nd | Nd | Nd | Nd | Neg |
| Xenodiagnosis | Nd | Nd | Neg | Pos | Pos | Neg |

nd: not done; na: does not apply.
